# Spontaneous Behaviors of Post-Orchiectomy Pain in Horses Regardless of the Effects of Time of Day, Anesthesia, and Analgesia

**DOI:** 10.3390/ani11061629

**Published:** 2021-05-31

**Authors:** Pedro Henrique Esteves Trindade, Marilda Onghero Taffarel, Stelio Pacca Loureiro Luna

**Affiliations:** 1 Department of Veterinary Surgery and Animal Reproduction, School of Veterinary Medicine and Animal Science, Sao Paulo State University (UNESP), São Paulo 18618-681, SP, Brazil; pedro.trindade@unesp.br; 2 Department of Veterinary Medicine, Maringa State University (UEM), Maringá 87502-970, Parana, Brazil; motaffarel@uem.br

**Keywords:** animal welfare, pain measurement, horses

## Abstract

**Simple Summary:**

Confounding factors may hinder the estimation of pain in horses. The current study aimed to identify spontaneous post-castration pain behaviors in horses regardless of the effects of anesthesia, analgesia, and recording time of day. Twenty-four horses divided into four groups were submitted to inhalation anesthesia only or combined with pre-operative analgesia, or castration under pre or postoperative analgesia. Thirty-four behaviors were evaluated in seven 60-min time-point recordings in the 24 h after anesthesia and at mirrored time-points in the 24 h before the anesthesia. Results showed changes in the behaviors of walk, look out the window, rest the pelvic limb, and rest standing still when assessed in the morning, afternoon, and night. The only pain-related behaviors observed regardless of the effects of time of the day, anesthesia, and analgesia, were a decrease in the mirrored proportional differences in time spent drinking and eating, and an increase in the mirrored proportional differences in the frequencies of look at the wound, retract the pelvic limb, expose the penis, and look at the back of the stall. In conclusion, confounding factors rather than pain may influence several suggestive pain behaviors documented in equine literature.

**Abstract:**

This prospective and longitudinal study aimed to identify spontaneous post-orchiectomy pain behaviors in horses regardless of the effects of anesthesia, analgesia, and recording time of day. Twenty-four horses divided into four groups were submitted to: inhalation anesthesia only (GA), or combined with previous analgesia (GAA), or orchiectomy under pre (GCA), or postoperative (GC) analgesia. The data obtained from the subtraction of frequency and/or duration of 34 behaviors recorded during seven 60-min time-points in the 24 h after the anesthesia from those recorded in the mirrored time-points in the 24 h before the anesthesia (delta) were compared over time and among groups by Friedman and Kruskal–Wallis tests, respectively (*p* < 0.05). Time of day influenced the behaviors of walk, look out the window, rest the pelvic limb, and rest standing still. The only pain-related behaviors were decreased mirrored proportional differences in time spent drinking, and eating, and increased mirrored proportional differences in the frequency or duration of look at the wound, retract the pelvic limb, expose the penis, and look at the back of the stall. In conclusion, confounding factors rather than pain may influence several suggestive pain-related behaviors documented in the literature.

## 1. Introduction

Several instruments have been proposed to assess acute pain in *Equus caballus* [1,2,3,4]. Behaviors related to activity, locomotion, appetite, attention to the environment, rest, and discomfort have been included in instruments to diagnose the pain of acute abdomen syndrome [5,6,7,8,9,10,11,12,13,14,15,16], dental procedures [16,17], orchiectomy [13,16,18,19], and orthopedic procedures [13,16,20,21,22,23,24,25]. The physiological parameters included in some of these instruments present questionable results due to their low specificity and sensitivity [1,10,14,20,23,25,26], added to which, they usually require the presence of the evaluator and a certain degree of intrusion [27]. Facial expression is a promising alternative for recognizing pain in horses [12,19,28], however, it requires experienced appraisers, specific training, and high-quality footage [1] or the observer’s presence [12]. The remote evaluation of body language using cameras, as in the case of pigs [29] and sheep [30], could also be reliable to assess pain in horses, as it is not invasive or intrusive [27,31,32].

Although efforts to develop a reliable and validated instrument to identify pain in horses are recognized, there is still no gold standard tool for this purpose [1,2,3,4,33,34]. Most studies evaluated acute pain after orthopedic surgery or celiotomy [5,6,7,8,9,10,11,12,13,14,15,16,17,18,19,20,21,22,23,24,25,35]. Only two studies assessed pain in hospitalized horses undergoing different painful conditions and intensities [13,16]. Some instruments were created in clinical situations where there was no possibility of controlling variables that can generate biases or confounders. One of these is the residual post-anesthetic effect of sedatives, anesthetics, and analgesics on behavior [7,12,15,21,26,36]. Moreover, on-site human evaluation is a common bias that may induce discomfort behaviors [27] or even inhibit both normal behavior expression before surgery and postoperative pain behaviors, as described in horses [27] and rabbits [37], suggesting that remote evaluation with a camera may be more appropriate to assess pain.

A confounding effect not yet studied in horses, and which could also interfere with pain assessment, is the period of the day in which the horse is evaluated. Throughout the day, the behavioral distribution is expected to be heterogeneous, for example, alert and active during the day and sleeping at night [38].

These biases could both maximize or minimize pain estimation and require investigation. To our knowledge, there are no studies in the literature on the effects of time of day on normal behavior and the implications of anesthesia, analgesia, and pain intensity on spontaneous behaviors of horses with acute postoperative pain, remotely assessed using a camera, without any human presence inside or in front of the stall. Therefore, the objective of this study was to identify the post-orchiectomy spontaneous pain behaviors in horses and the effects of anesthesia, analgesia, pain intensity, and time of day. The study hypothesis was that some spontaneous behaviors are related to acute postoperative pain, while others are confounded by the effects of anesthesia, analgesia, and recording time.

## 2. Material and Methods

The study is opportunistic of previous publications [18,39] approved by the institution’s Ethics Committee on the Use of Animals (ID186/2009) on 8 December 2009, and carried out with the consent of all owners. The experimental procedures presented in this study were developed in the previous publications [18,39]. In all groups the same periodical clinical examination and pain assessment were performed, and the data were published in the previous study [18]. The present study includes analysis of unpublished data from remote video recordings without visual contact or human presence inside or in front of the stalls.

### 2.1. Animals and Groups

Twenty-four crossbred horses, considered healthy after clinical and laboratory evaluations, were submitted to the same inhalation anesthesia protocol. Twelve experimental horses from the host institution not submitted to orchiectomy were randomly assigned to inhalation anesthesia only (GA *n* = 6; three geldings and three mares aged 9 ± 3 years and weighing 332 ± 48 kg) or inhalation anesthesia and analgesia (GAA *n* = 6; four geldings and two mares; 10 ± 5 years and 369 ± 68 kg). These institution-owned horses were familiar with the stalls, facilities and, personnel and the gelding horses from these groups had been submitted to previous intravenous anesthesia when they were castrated. Twelve client-owned horses who underwent elective orchiectomy were randomly treated with pre (GCA *n* = 6; 4 ± 2 years and 319 ± 48 kg) or postoperative analgesia (GC; *n* = 6; 4 ± 2 years and 302 ± 27 kg), to simulate two pain intensities (GC possibly representing severe pain and GCA possibly representing mild pain). These horses were having their first contact with the hospital stalls, facilities, personnel, and anesthesia procedures.

### 2.2. Management and Procedures

Twenty-four hours before the end of the anesthetic recovery, the horses were placed in individual stalls (4 × 4 m). Water and the same type of hay were available ad libitum for all horses before and after anesthesia when the horses were in the stall. The front stall door was divided into two parts. The superior half of the door (window) remained open to allow the horse to put its head out. None of the horses were subjected to preoperative fasting. The horses left the stall in the morning (07:43 ± 00:19 h) to be sedated with 0.5 mg/kg of xylazine (Sedomin^®^, König, Buenos Aires, Argentina) intramuscularly (IM). The preoperative analgesia for the GAA and GCA horses was 0.2 mg/kg of morphine (Dimorf^®^, Cristalia, Lindóia, Brazil) IM, 10 mg/kg of dipyrone (metamizole; Finador^®^, Ourofino, Brazil), and 1.1 mg/kg of flunixin meglumine (Desflan^®^, Ourofino, Cravinhos, Brazil) intravenously (IV) administered immediately before sedation. Anesthesia was induced in all horses with 100 mg/kg of guaifenesin 10% (EGG PPU Eter gliceril guaiacol^®^, LPS Agrofarma, Mogi Mirim, Brazil) and 5 mg/kg of thiopental IV (Thiopentax^®^, Cristalia, Lindóia, Brazil). After orotracheal intubation, the horses were hoisted onto a surgical table, positioned in dorsal recumbency, and general anesthesia was maintained with isoflurane (Isoforine^®^, Cristalia, Lindóia, Brazil) vaporized by oxygen (O_2_), under controlled ventilation (Mallard Medical Skypark Drive Redding^®^, United States) to maintain the anesthetic-surgical plane. Tidal volume was set at 15 mL/kg with a peak inspiratory pressure of 20 to 30 cm H_2_O to maintain normocapnia [end-tidal carbon dioxide (EtCO_2_) values between 4.66 and 6.00 kPa (35–45 mm Hg)]. Local anesthesia with 10 mL of 2% lidocaine with adrenaline (Lidocaina^®^, Cristalia, Lindóia, Brazil), injected into each spermatic cord before surgery, was performed only in GCA horses. The isoflurane vaporizer setting was adjusted by one anesthetist unaware of whether the animal had been treated (GCA) or not (GC) with pre-operative analgesia, to maintain an adequate anesthetic depth, confirmed by diminished, but not abolished, palpebral reflex, corneal reflex present, and stable heart rate and arterial blood pressure in response to surgical stimuli.

**Figure 1:**
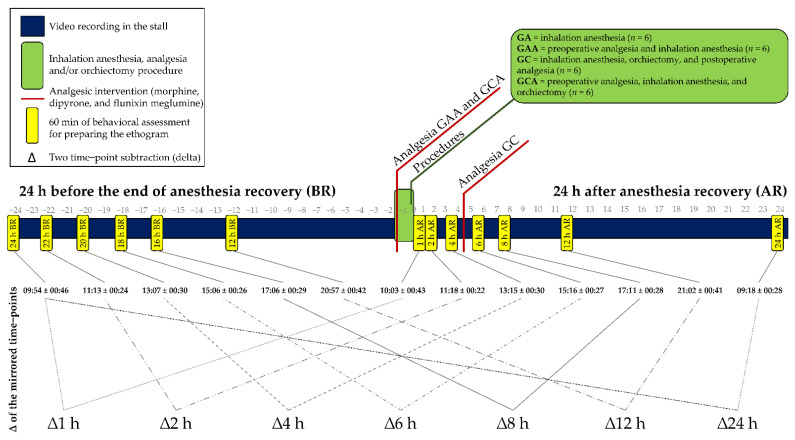
Timeline containing the 60-min behavior recording time-points, anesthetic, analgesic, surgical interventions, and identification of the mirrored time-points for extraction of the deltas. The delta (∆) is the subtraction of the frequency and duration of the behaviors recorded after the anesthesia from those recorded in the mirrored hours before the anesthesia.

Since none of the horses required anesthetic supplementation, it was assumed that they received equivalent amounts of anesthetic. Monitoring included heart rate, peripheral arterial hemoglobin saturation by pulse oximetry, inspired and end-tidal partial pressures of CO_2_, O_2_, and inspired and end-tidal isoflurane concentrations (Monitor Cardiocap 5^®^, DatexOhmeda, Helsinki, Finland). The facial artery was catheterized (20G, Introcan Safety^®^, Melsungen, Germany) to measure systolic, diastolic, and mean arterial blood pressure (MAP). The pressure transducer (TruWaveTM^®^, Edwards Lifesciences, San Cristobal, Dominican Republic), which was previously calibrated against a mercury column, was zeroed to atmospheric pressure and positioned at the right atrium level. When MAP was below 70 mm Hg, dobutamine (Dobutamina^®^, Biosintética Farmacêutica Ltd., São Paulo, Brazil) was administered intravenously as required, with a maximal dose of 5 μg/kg/min. Fluid therapy was provided with 5 mL kg/h of lactate ringer. The half-closed surgical castration technique was used [40]. The duration of anesthesia was approximately the same for all groups (48 ± 4 min), 45 ± 2 min, 51 ± 4 min, 50 ± 2 min, and 47 ± 3 min for GA, GAA, GC, and GCA, respectively. All horses recovered in a padded box with the lights on, without O_2_ supplementation, and were assisted with head and tail ropes when they attempted to stand up. After recovery from anesthesia and regaining the ability to stand, the horses were extubated. When the horses were able to move, they were guided to the same stall occupied before anesthesia where they remained for 24 h with free access to water and hay. Postoperative analgesia for GC horses was administered four hours and 20 min after anesthetic recovery, composed of morphine, dipyrone, and flunixin meglumine with the same dosage and route as described previously for GCA (Figure 1). Operated horses were treated with three administrations every 48 h of 30,000 IU/Kg of procaine penicillin G, benzathine penicillin G, and dihydrostreptomycin (Penfort^®^, Ourofino, Cravinhos, Brazil) IM.

### 2.3. Behavioral Assessment

The horses were filmed uninterruptedly for the 48 h in the stall, except during anesthesia and orchiectomy, using two video cameras, one fixed in the upper right corner in front of the stall and the other in the upper left corner at the back of the stall. Seven continuous time-points of 60 min without any human presence inside or in front of the stall were selected (0–1 h, 1–2 h, 3–4 h, 5–6 h, 7–8 h, 11–12 h, and 23–24 h) after recovery from anesthesia. The same six 60-min time-points recorded on the previous day (before anesthesia) were selected, corresponding to the same times as the after-anesthesia time-points (Figure 1). The time-points from 1 h to 24 h after the anesthesia were mirrored in the 24 h before the anesthesia. An extra time-point was also recorded between 2 and 3 h before anesthesia (3 h BR; 06:43 ± 00:32) without a mirrored record, which was considered a baseline in a previous study [18].

An ethogram was developed by including the relevant behaviors to assess acute postoperative pain based on previous studies [1,7,11,12,20,21,22,36,41] and our recordings. One of the authors of the study (MOT) continuously recorded the frequency of 18 events (behaviors with shorter duration) and duration in minutes of 16 states (longer-lasting behaviors) of 34 behaviors divided into 9 categories according to pain diagnostic criteria reported in previous studies [7,11,12,18,20,22,23,36,41] (Table 1). The focal animal sampling method was used by observing and recording the frequency and duration of each horse’s behaviors for a continuous period of time [42]. The evaluator was the same for all observations and was blinded to the GA vs. GAA and GC vs. GCA.

### 2.4. Statistical Analysis

All statistical analyses were performed by PHET in software R using the integrated development environment RStudio (Version 4.0.2 (2020−06−22), RStudio, Inc. Boston, MA, USA). The functions and packages were presented in the format “function {package}” and α was considered 5% in all analyses. The data distribution was investigated using histograms (hist {graphics}), quantile-quantile graphics (qqnorm {stats}), and boxes (ggboxplot {ggpubr}). According to the graphs, no variables were normally distributed. The behavior, expose the penis, was considered missing data for females.

Intergroup comparisons were performed to infer the effects of anesthesia only (GA), pre-operative analgesia and anesthesia (GAA), preoperative analgesia, anesthesia, and orchiectomy (GCA) possibly representing mild pain, and anesthesia, orchiectomy, and postoperative analgesia (GC), possibly representing severe pain. The analysis was based on the delta (Δ) extraction of the frequency and duration of each behavior by subtracting the values at each time-point after the anesthesia from its mirrored time-point at equivalent times before the anesthesia for each horse (Figure 1). Intergroup comparisons were investigated at each time-point using the Kruskal–Wallis test (kruskal {agricolae}) with multiple post-test comparisons with the false discovery rate. Delta results show the proportional increase or decrease in frequency or duration of each behavior recorded at each time-point in relation to the same time recorded the day before the anesthesia. The criterion for determining the effect of anesthesia, analgesia, and pain on behavior was based on differences between GC and the other groups for each time-point.

To investigate the effect of the recording time, alterations in behavior on the day before the anesthesia were analyzed using the Friedman test (friedman.test {stats}) with multiple post-test comparisons based on the false discovery rate (posthoc.friedman.nemenyi.test {PMCMR}). For this stage, behavior frequency, and duration, and not the delta, were used for all groups combined, because all horses were under the same conditions and had not yet undergone any procedure.

An extra analysis was conducted only with the raw data of frequency or duration of the six pain-related behaviors unaffected by the time of day and by the effects of anesthesia and analgesia. The Friedman test was used for within-group comparisons over time and the Kruskal–Wallis test with multiple post-test comparisons based on the false discovery rate was used for inter-group comparisons at each time point. The baseline time-point adopted was that used in the previous publication [18] (3 h BR) and following the time-points subsequences after anesthesia (1, 2, 4, 6, 8, 12, and 24 h AR).

## 3. Results

There were no differences in Δ between groups for any time-point for the following behaviors: flehmen, head below withers, kick, lateral decubitus, lower head, masticatory movements, movements with the tongue, paw, raise the tail, retract and extend pelvic limb, roll, scratch, shake, smell, stare at the side of the stall, sternal decubitus, stretch the body, swing tail, try to lie down, urinate, vertical movement of the head, and yawn. This means that the expression of these behaviors did not vary from the same time of the day before anesthesia in any groups, and these behaviors were not affected by analgesia, anesthesia, or surgery, and possibly are not associated with pain.

**Figure 2:**
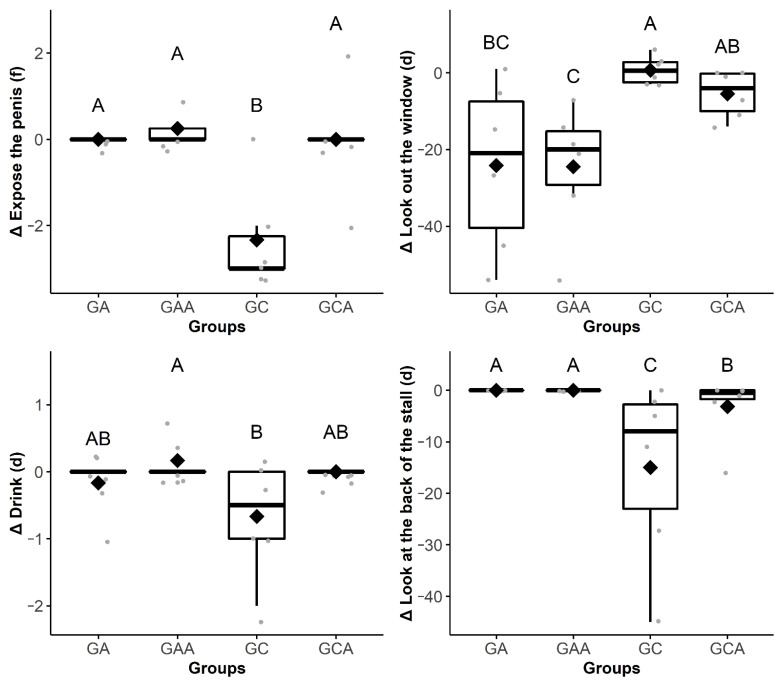
Boxplot of Δ1 h (10:03 ± 00:43) showing the differences in the frequency (^f^) or duration (^d^) of the behaviors expose the penis ^f^, drink ^d^, look out the window ^d^, and look at the back of the stall ^d^ among the groups of horses submitted to anesthesia (GA), previous analgesia and anesthesia (GAA), anesthesia, orchiectomy, and postoperative analgesia (GC) and preoperative analgesia, anesthesia, and orchiectomy (GCA). The delta (∆) is the subtraction of the frequency or duration of the behaviors recorded after the anesthesia from those recorded in the mirrored hours before the anesthesia. Different uppercase letters indicate a significant difference over time (A > B > C). The gray dots represent each horse; “d” indicates behaviors assessed as duration and “f” as frequency.

**Figure 3:**
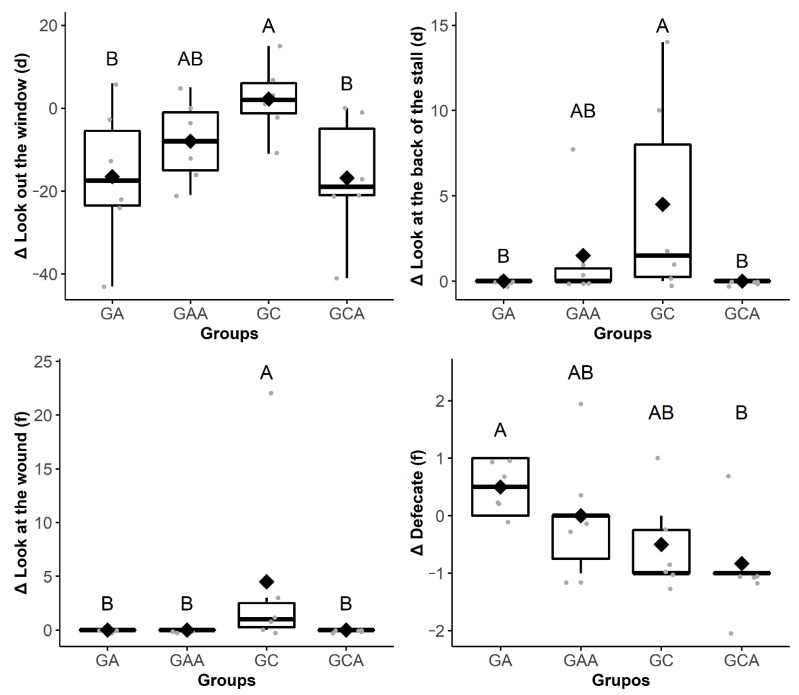
Boxplot Δ2 h (11:18 ± 00:22) showing the differences in the frequency (f) or duration (d) of the behaviors look out the window^d^, at the back of the stall^d^, and at the wound^f^, and defecate^f^ among the groups of horses submitted to anesthesia (GA), preoperative analgesia and anesthesia and analgesia (GAA), anesthesia, orchiectomy, postoperative analgesia (GC) and preoperative analgesia, anesthesia, orchiectomy (GCA). The delta (∆) is the subtraction of the behaviors frequency or duration recorded after the anesthesia from those recorded in the mirrored hours before the anesthesia. Different uppercase letters indicate a significant difference over time (A > B). The gray dots represent each horse.

**Figure 4:**
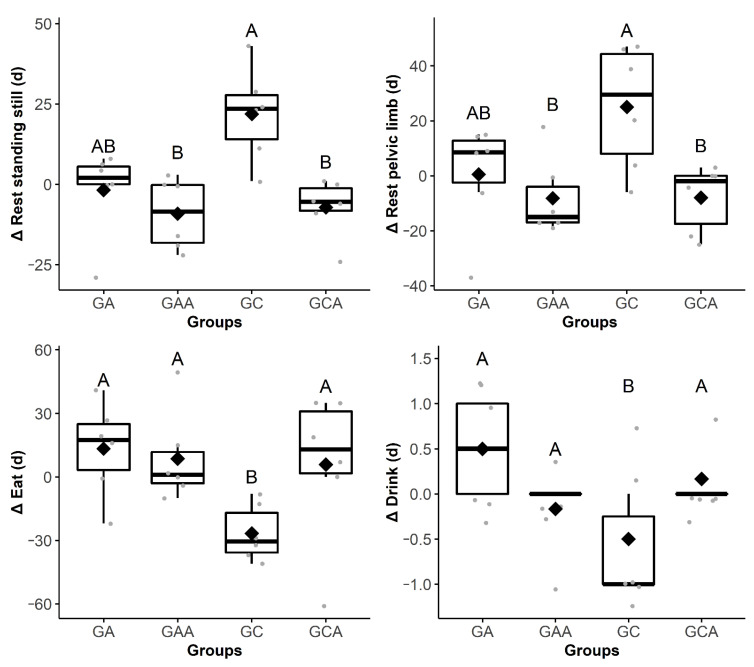
Boxplot Δ4 h (13:15 ± 00:30) showing the difference in the duration (d) of the behaviors rest standing still, eat, rest pelvic limb, and drink among the groups of horses submitted to anesthesia (GA), preoperative analgesia and anesthesia (GAA), anesthesia, orchiectomy, and postoperative analgesia (GC) and preoperative analgesia, anesthesia, and orchiectomy (GCA). The delta (∆) is the subtraction of the behaviors frequency or duration recorded after the anesthesia from those recorded in the mirrored hours before the anesthesia. Different uppercase letters indicate a significant difference over time (A > B). The gray dots represent each horse.

**Figure 5:**
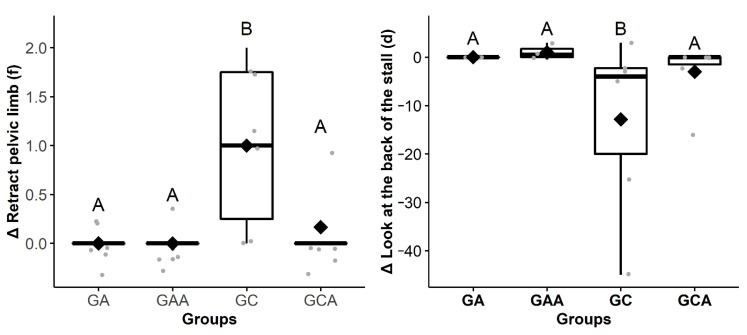
Boxplot Δ24 h (09:18 ± 00:28) showing the difference in the frequency (f) or duration (d) of the behaviors retract pelvic limb^f^ and look at the back of the stall^d^ among the groups of horses submitted to anesthesia (GA), anesthesia and analgesia (GAA), preoperative analgesia and anesthesia, orchiectomy, and postoperative analgesia (GC) and preoperative analgesia, anesthesia, and orchiectomy (GCA). The delta (∆) is the subtraction of the behaviors frequency or duration recorded after the anesthesia from those recorded in the mirrored hours before the anesthesia. Different uppercase letters indicate a significant difference over time (A > B). The gray dots represent each horse.

Horses from the GC exhibited proportionally increased frequency of behaviors of look at the wound (Δ2 h) and retract the pelvic limb (Δ24 h) compared to the other groups, while duration or frequency of drink (Δ4 h), eat (Δ4 h), walk (Δ6 h), look at the back of the stall (Δ1 h), and expose the penis (Δ1 h) were proportionally decreased in comparison with the other groups (Table 2 and Figure 2, Figure 3, Figure 4 and Figure 5). The raw data of frequency or duration of the behaviors that showed Δ differences between groups (Table 2) are presented in Table S1. The intra and inter-group differences in frequency or duration of behaviors not affected by time of day, anesthesia, and analgesia are expressed in Table S2. Only the behaviors look at the wound and retract the pelvic limb showed similar differences compared to the analysis based on Δ (Table 2).

When evaluating the effect of the recording time before the anesthesia, the horses walked and kept their heads out the window for less time and rested standing still and rested the pelvic limb for longer during the night, in relation to the daytime (Table 3). Table 4 combines the behaviors associated only with pain (and not affected by anesthesia and analgesia) and those affected by the time of the day (before anesthesia) in the current study and in studies available in the literature.

## 4. Discussion

The novelty of the present study was the calculation of the Δ between mirrored post- and pre-anesthesia time-points to verify the most relevant behavior changes between horses submitted only to anesthesia or anesthesia and orchiectomy with or without analgesia in both cases. To our knowledge, this study is pioneering in identifying frequency and/or duration of spontaneous behaviors without any human presence inside or in front of the stall to assess acute post-orchiectomy pain in horses, considering the interference of anesthesia, analgesia, pain intensity, and recording time. Our findings suggest that the behaviors drink, eat, look at the back of the stall, look at the wound, retract the pelvic limb, and expose the penis are probably pain-related behaviors, independent of the time of day and the effects of anesthesia and analgesia. Walk, rest standing still, look out the window, and rest pelvic limb were relevant to the diagnosis of pain in previous studies [5,6,7,8,9,10,11,15,16,18,19,20,22,36], however, because they were influenced by the time of day in the present study, the observation time may be a confounding factor when these behaviors are used to assess postoperative pain in horses. On the other hand, other behaviors suggestive of pain in the literature (flehmen, head below withers, kick, lateral decubitus, lower head, paw, retract and extend pelvic limb, roll, scratch, shake, stare at the side of the stall, sternal decubitus, stretch the body, swing tail, try to lie down, urinate, vertical movement of the head, and yawn) [10,11,12,18,21,22,23,36] were not modified, suggesting that they are not associated with pain after orchiectomy. It was impossible to differentiate mild pain in castrated horses treated with preoperative analgesia (GCA) from severe pain in horses not treated with preventive analgesia (GC).

The time spent looking out the stall window in the immediate postoperative period in surgical horses not treated with preoperative analgesia was proportionally less affected compared to the other groups and may represent a confounder to assess pain in this period. Lack of interest in the environment is a possible indicator of pain [10,11,18,23], however, according to our results, caution should be taken when interpreting this behavior immediately after anesthetic recovery. Surprisingly, contrary to expectations for instruments assessing pain [11,12,17,18,20,21,22,28], at the first hour after recovery, the Δ duration of look out the window was not altered in the castrated horses, otherwise it was proportionally decreased in the other groups. This discrepancy could be related to the fact that in previous studies evaluations were not carried out immediately after anesthetic recovery. A possible explanation for the proportionally greater attention to the environment in surgical horses, compared to those only anesthetized, is anxiety as a non-specific response due to pain [36] or dislocated behavior, as reported in neonatal dogs after caudectomy without analgesia, who suckled more than those who received pain relief [43]. Another possible explanation is that the horses only submitted to anesthesia were from the host institution and were less curious, probably because they were more familiar with the environment. This result requires further investigation.

Although at the anticipated time-point of maximum pain (Δ4 h) [18], changes in various behaviors were expected, only the time spent eating and drinking proportionally decreased in horses submitted to surgery without previous analgesia compared to the other groups. As expected, horses receiving preventive pain relief from the analgesics and those only anesthetized ate proportionally more than horses without pain relief. Once pain relief was provided to the post-analgesia surgical group, eating behavior was like the other groups. In addition to pain relief, morphine increases food intake due to a direct effect of the opioid on neural mechanisms controlling feeding [44]. The importance of eating for pain assessment may be disputable because two of six horses ate even before analgesia, while two horses ate at 1 h and the other two ate only 4 h after analgesia. In this study, horses had continuous access to hay and were not fasted before anesthesia. Preoperative fasting is a possible confounding factor for interpreting the importance of appetite in pain assessment, since, after starvation, even horses experiencing pain tend to eat after surgery. Other confounders include variations in the routine of food offer, types, and quality, and individual preferences. Although pre-anesthetic starvation is a common practice in horses, the benefits of this procedure are disputable [45]. We decided not to deprive horses of food to avoid a possible effect of starvation increasing appetite after anesthesia.

Based on the Δ differences in the pain-related behaviors at different time-points (proportionally greater mirrored frequency of the behaviors look at the wound and retract the pelvic limb and proportionally smaller duration and/or frequency of drink, eat, look at the back of the stall, and expose the penis), only the last was not included in previously published pain scales [7,9,11,18,22]. These differences using the Δ approach for statistical analysis were not observed when the raw data of frequency and duration were used, suggesting that this method enhanced the possibility of detecting changes in behavior in response to pain, despite the trade-off of requiring more effort to understand the findings. In the present study, there were no expected significant alterations in possible pain behavior indicators reported in other studies [10,11,12,16,18,21,22,23,41] (flehmen, head below withers, kick, lateral decubitus, lower head, paw, retract and extend pelvic limb, scratch, shake, stare at the side of the stall, sternal decubitus, stretch the body, swing tail, urinate, vertical movement of the head, movements with the tongue, and yawn). The absence of changes in these behaviors, except roll and try to lie down, which are specific to abdominal pain [5,6,7,8,9,10,11,12,13,14,15,16], may be explained because (i) the pain model used in the present study is not expected to produce intense pain, and/or (ii) the behaviors are specific to a particular type of pain but do not occur in other conditions, and/or (iii) they are possibly irrelevant for diagnosing pain, and/or (iv) the sample number was small. Some other behaviors (masticatory movements, smell, and raise the tail) that we suspected would be associated with pain did not show changes in this or previous studies [1,2,3,4,5,6,7,8,9,10,11,12,13,14,15,16,17,18,19,20,21,22,23,24,25,36,41] and may not be important to diagnose pain.

This study mimicked a real scenario by comprising the usual transit of people during the clinical routine of a veterinary hospital. In all horses the same periodical clinical examination and pain assessment, including interaction with the observer, locomotion when led by the evaluator, and palpation to the affected area were performed and the data published in the previous study [18]. However, considering that human presence disturbs discomfort behavior in confined horses [27], in this study the recorded data were obtained remotely by video cameras, only when there was no presence of any human at the front of the door or inside the stall where the horse was housed. Our findings suggest that it is possible to diagnose post-orchiectomy pain based only on spontaneous behaviors, questioning the need for direct human pain assessment interventions [7,10,12,15,18,21,22].

Our results suggest that some adjustments could be performed in previously published pain assessment instruments to define the more specific pain behavioral indicators, as previously reported for pigs [29], sheep [30], cats [46], and oxen [47]. Except for the visceral pain scale for orchiectomy [18] and the orthopedic scale [22], other scales [6,8,9,12,21] did not consider the isolated analysis of the importance of each behavior according to internal consistency, item-total correlation, principal components analysis, sensitivity, and specificity, possibly leading to the inclusion of unnecessary behaviors in these instruments [48].

The quantification of pain is relevant to identify various degrees of suffering and aid decision-making for qualitative selection and dosage of analgesic intervention. Although it was expected that horses castrated with prior analgesia would express an intermediate degree of pain between those castrated without previous analgesia and those only anesthetized, this was not observed even 8 h after the anesthesia, when the analgesic effect of the morphine (2 h) [49], dipyrone (5 h) [50], and flunixin meglumine (8 h) would be fading [51]. It can be inferred that the analgesic protocol produced preventive analgesia. Horses treated with different quality analgesic protocols also did not show differences in the orthopedic composite behavioral scale after castration [19]. It is important to assess mild or moderate pain in practice to evaluate whether the horse requires analgesia or whether analgesia was adequate. The challenge of recognizing pain of mild or moderate intensity requires further investigation. In other species, this challenge has been unraveled by the definition of a cut-off point for rescue analgesia [29,30,46,47].

The second innovation of this study was the use of mirrored time-points, to control and assess the effect of the time of day on behavioral records 24 h before and after the interventions. This strategy identified the time-dependent behaviors before the anesthesia, as reported in rabbits [52]. The behaviors walk, rest standing still, look out the window, and rest pelvic limb have previously been applied to assess pain in horses [5,10,11,12,18,20,22,23], yet, according to our results, they are influenced by the time of day. Naturally, behaviors change throughout the day; horses are alert and active during the day and resting or sleeping at night [38]. These behavioral variations may also be related to greater movement by hospital staff in the daytime compared to at night. The practical implication of these findings is that it is advisable to relativize the importance of or disregard these behaviors to avoid false-positive results in interpreting pain intensity at night. Despite this, most behaviors pointed out in this and previous studies [9,10,11,12,18,21,22,23] did not suffer interference from the time of day.

Study limitations include the differences in numbers of each sex, between only male horses undergoing orchiectomy from animals that were only anesthetized, composed of castrated females and males. The normal daily behavior of stallions and free-living mares is different [53]. The estrous cycle influences the aggressive behavior (kicking, bucking, biting) in mares when handled [54], while stallions are more curious to an enriched environment in comparison to castrated and female horses [55]. In our study, except for the attention to the environment, the pain-related behaviors were related to feeding, relaxing, and attention to the affected area, which we believe suffer less sex influence. Although exposing the penis is unavailable in females, it is relevant for assessing pain, because time of the day, anesthesia, and analgesia did not influence exposing the penis and after surgery males castrated with postoperative analgesia exposed the penis proportionally less than those castrated with preoperative analgesia. Another limitation was that the non-operated horses, but not the operated ones, were familiar with the hospital environment because they belonged to the institution; therefore, the horses submitted to orchiectomy had no prior acclimatization to the hospital. Lack of adaptation to the environment reflects the real-life circumstances of an active equine hospital but may deflagrate curiosity and increase restlessness. New arena tests (open field) investigating fear or curiosity in a new environment are usually conducted during the first 20 min of horse exposure to a new environment [56], therefore it seems that the most important reactions occur for a short time. To partially overcome this limitation, the current study showed that behaviors related to environment familiarity, like look out the window, walk, and rest standing still, were different even between the groups of castrated horses with or without previous analgesia, that were equally acquainted with the surroundings, showing that they may be considered pain-related behaviors and are not only associated with environment adaptation.

The practical implications of this study are that the pain-related spontaneous behaviors identified may be used as a basis for future studies to build a shorter and simpler composite behavioral pain scale compared to those available to date [7,12,13,15,16,17,18,19,21,22], in order to reduce assessment time in a busy hospital routine. Our findings contribute to the avoidance of confounding factors and to guaranteeing a more accurate diagnosis of pain in this species.

The authors recognize that behavioral-based pain assessment is one of the best approaches to diagnose equine pain in the clinical and experimental setting [7,12,13,15,16,17,18,19,21,22]; nevertheless, future studies should consider that time of the day, anesthesia, and analgesia affect the frequency and/or duration of the pain-related spontaneous behaviors. Our results were based on 60 min periods of observation; prolonged observation periods may be necessary to identify the relevant, exclusive, and subtle equine pain behavioral changes [41]. Perhaps the current paradigm that it is possible to perform in-person equine pain assessment for up to 10−15 min demands further investigation. Artificial intelligence-based tools might help us to collect and analyze data automatically to build and implement a robust, reliable, and valid instrument in the clinical and experimental setting, to include detection of mild and severe pain.

## 5. Conclusions

Because the spontaneous behaviors drink, eat, look at the back of the stall, look at the wound, retract the pelvic limb, and expose the penis were not affected by anesthesia, analgesia, or time of the day, they may be considered pain-related behaviors and have the potential to be used as post-orchiectomy pain indicators in horses under remote video evaluation without human intervention. Because the time of day may be a confounding factor for the behaviors walk, rest standing still, look out the window, and rest pelvic limb, their importance should be relativized according to the time they are evaluated. Other behaviors considered suggestive of pain (flehmen, head below withers, kick, lateral decubitus, lower head, paw, retract and extend pelvic limb, scratch, shake, stare at the side of the stall, sternal decubitus, stretch the body, swing tail, urinate, vertical movement of the head, movements with the tongue, and yawn) were not significantly modified in horses submitted to orchiectomy and require further scrutiny in horses suffering pain under other conditions. No behaviors defined the effect of pain intensity.

## Figures and Tables

**Table 1:** Description and category of the recording of 34 spontaneous behaviors classified in 9 categories to compose the ethogram of 24 horses after anesthesia, analgesia, and orchiectomy (^d^ duration; ^f^ frequency). The behavioral categories were based on previous studies [7,11,12,18,20,22,23,36,41].

Category	Spontaneous behaviors	Description
Feeding	Drink ^d^	Immerse part of the mouth in the water in the drinking trough, suck, and swallow water.
	Eat ^d^	Hold the hay with the lips, chew, and swallow.
Attention to the affected area	Swing tail ^f^	Move the tail left or right, or at least 45° upwards, and then back to the starting position.
	Kick ^f^	Retract the pelvic limb quickly, flexing the tarsal joint to strike the abdomen, and the hoof may touch the abdomen.
	Retract pelvic limb ^f^	Retract the pelvic limb, flex the tarsal joint without touching the hoof on the abdomen, and keep the retracted limb in suspension for a few seconds before returning to the initial position.
	Retract and extend pelvic limb ^f^	Retract the pelvic limb, flexing the tarsal joint, stretch the limb back, and return the limb to the starting position.
	Look at the wound ^f^	Direct the head and muzzle towards the inguinal region (groin).
Attention to the environment	Smell ^f^	Touch the muzzle to a structure in the stall, inhaling, and exhaling air (smelling).
	Flehmen ^f^	Raise the head forward, contracting the upper lip upwards towards the nostrils.
	Look at the back of the stall ^d^	Stand in the middle of the stall with the head facing away from the stall door.
	Look out the window ^d^	Stand in front of the stall door with the head positioned outside the stall through the window.
	Stare at the side of the stall ^d^	Stand at the back or middle of the stall with the head facing one side of the stall.
	Stay at the back of the stall ^d^	Stand on the opposite side from the stall door (back of the stall), however, with the head directed towards the stall door.
Self-care	Scratch ^f^	Turn the neck to one side towards the body, lean the muzzle against the body, and scratch the body surface with movements of the upper lip (muzzle) or quick bites.
Rest	Lower head ^f^	Move the upper edge of the head (occipital) down the withers.
	Head below withers ^d^	Keep the upper edge of the head (occipital) below the withers.
	Rest standing still ^d^	Stationary with the four members on the floor supporting the body weight (standing).
	Rest pelvic limb ^d^	Standing supporting the bodyweight with three limbs resting on the floor, while one of the pelvic limbs remains relaxed, with no load and may have only the hoof tip resting on the floor.
Discomfort	Try to lie down ^f^	Lower the head, smell the floor, and may or may not flex the thoracic limbs and touch the floor (kneeling), and return to the quadrupedal position.
	Yawn ^f^	Open the mouth with the head extended forward and rotate the jaw before closing the mouth.
	Raise the tail ^d^	Raise the tail’s base upwards more than 45º and keep it raised for a few minutes and return to the starting position.
	Paw ^f^	Raise one of the thoracic limbs forward, lean the hoof on the floor in front of the body, and drag the hoof across the floor until it returns to the initial position close to the body.
	Sternal decubitus ^d^	Lie with the sternum resting on the floor.
	Lateral decubitus ^d^	Lie with ribs resting on the floor.
	Movements with the tongue ^f^	Put tongue out of the mouth and lick lips without eating or drinking water.
	Vertical movement of the head ^f^	Move head up and down at least once.
	Masticatory movements ^d^	Distance the mandible from the maxilla (chewing), open the mouth without eating or drinking water.
	Roll ^f^	When lying on the floor in lateral decubitus move the limbs, neck, and head from side to side.
Excretion	Defecate ^f^	Raise the tail and defecate.
	Urinate ^f^	Eliminate a stream of urine.
Locomotion	Walk ^d^	Move the four limbs at least once forward, backward, or sideways, changing position.
Relax	Expose the penis ^d^	Completely expose the penis out of the prepuce.
	Stretch the body ^f^	Stand supporting the bodyweight on the four limbs, stretch the neck down or forward, arching the spine upward, stretching.
	Shake ^f^	Rotate the head, neck, and upper body quickly and rhythmically.

**Table 2:** Median and interquartile range (Q_1_; Q_3_) of the delta (Δ) of the frequency or duration of the behaviors that showed differences between groups of horses submitted to anesthesia (GA), previous analgesia and anesthesia (GAA), anesthesia, orchiectomy, and postoperative analgesia (GC) and preoperative analgesia, anesthesia, and orchiectomy (GCA). The delta (∆) is the subtraction of the frequency and duration of the behaviors recorded after the anesthesia from those recorded in the mirrored hours before the anesthesia. Different uppercase letters and values in bold indicate a significant difference between groups at each time-point (A > B > C). The vertical dashed black line indicates when postoperative analgesia was performed for the GC; ^d^ indicates behaviors assessed as duration and ^f^ as frequency.

Behaviors	Groups	Time-Points						
		∆1 h	∆2 h	∆4 h	∆6 h	∆8 h	∆12 h	∆24 h
Drink ^d^	GA	0 ^AB^ (−0.2; 0)	0 (−0.2; 0.2)	0.5 ^A^ (0; 1)	0 ^AB^ (−0.2; 0.2)	0 (0; 0.2)	0 (−0.2; 0.2)	0 (−1; 1)
	GAA	0 ^A^ (0; 0.2)	−1 (−1; −0.7)	0 ^A^ (−0.2; 0)	1 ^A^ (0; 1)	0 (0; 1)	0 (−0.2; 0.2)	0 (0; 1.2)
	GC	−0.5 ^B^ (−1.2; 0)	−0.5 (−2.5; 0)	−1 ^B^ (−1; 0.2)	−1 ^B^ (−1; 0)	0 (−1.2; 0.5)	−0.5 (−1.2; 0.2)	1 (0; 1.2)
	GCA	0 ^AB^ (0; 0)	−0.5 (−1; 0)	0 ^A^ (0; 0.2)	0 ^B^ (−0.2; 0)	0 (0; 1)	0 (−0.2; 0.2)	0 (0; 0.2)
Eat ^d^	GA	−3.5 (−11.7; 22)	10.5 (−11.7; 32)	17.5 ^A^ (−6.2; 30.5)	−7 (−11.2; 28.2)	−2 (−18.5; 11.2)	4 (−9; 9.5)	8.5 (−27.7; 26)
	GAA	−1.5 (−17; 14)	−9 (−18; 8.7)	1 ^A^ (−5.5; 23.5)	13.5 (−5.5; 20.2)	7.5 (−2.7; 26.7)	−5 (−19.2; 12.5)	−4.5 (−26; 6.5)
	GC	−11.5 (−28; −6)	−14.5 (−34.5; 0.5)	−30.5 ^B^ (−38; −11.7)	15 (−7.5; 22.7)	16.5 (5; 26.7)	1 (−12.5; 23.5)	5 (−10.5; 21.7)
	GCA	−6.5 (−19; 19.7)	25.5 (−23.7; 31.2)	13 ^A^ (−15.2; 35)	1 (−15.7; 10.2)	−22.5 (−36.2; 26.5)	−9 (−20.2; 18.7)	−8 (−13; 7.7)
Defecate ^f^	GA	0 (−1; 1.2)	0.5 ^A^ (0; 1)	0.5 (0; 1)	0.5 (0; 1)	0 (−1; 0)	0 (−0.2; 1)	0 (−0.2; 1.2)
	GAA	−1 (−1.2; 0)	0 ^AB^ (−1; 0.5)	−1 (−1.2; 0)	0 (−0.2; 0.5)	0 (−3; 1.5)	0.5 (0; 1.2)	0 (−1; 1.2)
	GC	0.5 (−1; 1)	−1 ^AB^ (−1; 0.2)	−1 (−1.2; 0.2)	0 (−1; 0)	−1 (−1.5; 0)	0 (−1; 0.5)	0 (0; 1)
	GCA	−1 (−1; 0.2)	−1 ^B^ (−1.2; −0.5)	0 (−1; 0.5)	0 (−0.2; 0.2)	0 (−1; 0.2)	0 (−1; 0.2)	−0.5 (−1; 1)
Walk ^d^	GA	0 (−2.7; 1)	0 (−0.5; 2.2)	1.5 (−1.5; 4)	2.5 ^A^ (0; 6.2)	−1.5 (−4.7; 1.5)	0 (−2.5; 0.5)	1 (−0.2; 4.5)
	GAA	0 (−1; 0)	0.5 (−0.2; 5)	3 (0.2; 6.7)	1.5 ^A^ (0.7; 4.2)	1 (−1; 2.2)	1 (0; 1.2)	2.5 (0.7; 9.2)
	GC	0 (−0.2; 1)	0.5 (−0.5; 2.5)	0 (−1.2; 0.5)	−1 ^B^ (−2; −1)	0 (−2.5; 1)	0.5 (−0.2; 1.5)	0 (0; 0.5)
	GCA	−1 (−2.5; 0.2)	0 (−2; 7)	0.5 (−1.7; 2.5)	1 ^A^ (0; 3.2)	0.5 (−1.5; 2)	0 (−0.2; 1.2)	0.5 (−2.5; 2.2)
Rest standing still ^d^	GA	0 (−0.5; 0)	0 (−3.7; 0.2)	2 ^AB^ (−7.2; 6.5)	0 (−7; 5.2)	1.5 (−4.7; 5.5)	−9.5 (−20; 3.7)	0 (−0.5; 0.2)
	GAA	0 (−12.7; 0)	0 (−9.5; 2.7)	−8.5 ^B^ (−19.7; 0.7)	−7 (−14.5; 0.2)	0 (−0.7; 1.5)	−9.5 (−24.5; 14.7)	0.5 (−6.2; 3)
	GC	−14 (−25.5; 2.7)	7.5 (−2.5; 16.2)	23.5 ^A^ (8.5; 32.5)	−12.5 (−19; 8.7)	−8 (−13.2; 1.2)	2 (−33.5; 13.5)	−2 (−13.7; 7.5)
	GCA	−8 (−22.2; 0)	−4.5 (−16; 0.2)	−5.5 ^B^ (−12.7; 0.2)	2.5 (−13.7; 12.5)	0 (−3.7; 4)	4 (−3.2; 17.2)	1.5 (−7.7; 17)
Stay at the back of the stall ^d^	GA	0 (0; 0)	0 (0; 0)	0 (−0.5; 0)	0 ^A^ (0; 1.7)	0 (−0.2; 1.5)	0 (0; 0.2)	0 (0; 0)
	GAA	0 (0; 0)	0 (−0.2; 0.5)	0 (0; 0)	0 ^AB^ (0; 0)	0 (−0.5; 0)	0 (0; 0)	0 (0; 0.2)
	GC	0 (−20.5; 0)	0 (−1; 0)	0 (−2.2; 1.7)	−2 ^C^ (−7.5; 0)	0 (0; 1)	0 (−0.7; 4)	0 (−14.2; 13)
	GCA	0 (−9.2; 0)	0 (−1.7; 0)	0 (−3.5; 0)	0 ^BC^ (−9.2; 0)	0 (0; 0.2)	1 (0; 5.5)	0 (−4; 3.7)
Look out the window ^d^	GA	−21 ^BC^ (−47.2; −3.5)	−17.5 ^B^ (−28.7; −0.7)	−10 (−21; 11)	−1 (−23.7; 10.7)	4 (−2; 11)	1.5 (−5.2; 6.7)	−7.5 (−29.2; 27.7)
	GAA	−20 ^C^ (−37.5; −12.2)	−8 ^AB^ (−17.2; 1.2)	3 (−15.7; 18)	−4.5 (−13.5; 5.5)	−2 (−24.7; 4.5)	6 (0.7; 12)	0.5 (−16.2; 21)
	GC	0.5 ^A^ (−3; 3.7)	2 ^A^ (−4.2; 9)	1.5 (−3.7; 8.2)	−1.5 (−10; 1.2)	−0.5 (−11; 2.7)	0.5 (−2.5; 5)	2.5 (0.5; 6.2)
	GCA	−4^AB^ (−11.7; 0)	−19 ^B^ (−26; −0.7)	−5.5 (−22; 1.5)	6.5 (−7.2; 12.5)	1.5 (−9; 16)	2.5 (0; 11.7)	6 (1; 13)
Look at the back of the stall ^d^	GA	0 ^A^ (0; 0)	0 ^B^ (0; 0)	0 (0; 0)	0 (0; 0.2)	0 (−1.7; 0)	0 (−10.7; 0.2)	0 ^A^ (0; 0)
	GAA	0 ^A^ (0; 0)	0 ^AB^ (0; 2.7)	0 (0; 0)	0 (−0.5; 2.2)	0 (−0.2; 0)	0 (0; 0)	0.5 ^A^ (0; 2.2)
	GC	−8 ^C^ (−31.5; −1.5)	1.5 ^A^ (0; 11)	0 (−2.5; 19)	−0.5 (−15.7; 0.2)	0 (−11.2; 0)	2.5 (0; 9.7)	−4 ^B^ (−30; −0.7)
	GCA	−0.5 ^B^ (−5.5; 0)	0 ^B^ (0; 0)	0 (−1.2; 0)	0 (−1.2; 0.2)	0 (0; 1)	0 (0; 0.7)	0 ^A^ (−5.5; 0)
Look at the wound ^f^	GA	0 (0; 0)	0 ^B^ (0; 0)	0 (−0.5; 0)	0 (−0.5; 0)	0 (0; 0)	0 (0; 1.2)	0 (0; 0)
	GAA	0 (0; 0)	0 ^B^ (0; 0)	0 (0; 0)	0 (0; 0)	0 (0; 0)	0 (0; 0)	0 (0; 0)
	GC	0 (−0.2; 3.5)	1 ^A^ (0; 7.7)	0 (0; 3.5)	0 (0; 2.7)	0 (0; 1.2)	0 (0; 0.5)	0 (−0.5; 0)
	GCA	0 (0; 0)	0 ^B^ (0; 0)	0 (0; 2.7)	0 (0; 0.5)	0 (0; 0)	0 (0; 0)	0 (0; 0)
Rest pelvic limb ^d^	GA	−11 (−19.5; 0.7)	−4.5 (−19; 3)	8.5 ^AB^ (−13.7; 14.2)	2 (−20.2; 9.2)	5.5 (0.5; 10.2)	4.5 (−11.5; 13.5)	−7.5 (−19.7; 4.2)
	GAA	−18 (−35; 0)	−5.5 (−19.5; 1.2)	−15 ^B^ (−17.5; 3.7)	−15 (−20.2; −10.7)	0 (−4.2; 5.2)	−3 (−20.5; 13.2)	−11 (−20.2; 0.2)
	GC	6 (−18; 9.2)	14 (−3; 25)	29.5 ^A^ (1.5; 46.2)	0.5 (−11.7; 8)	−3.5 (−11.2; 0)	−2 (−35.2; 3.2)	−13 (−22.7; 2)
	GCA	−0.5 (−15; 0)	−0.5 (−2.7; 0.2)	−2 ^B^ (−22.7; 0.7)	2.5 (−13; 6.5)	−2 (−6; 5.5)	1.5 (−5.2; 15.2)	−0.5 (−3.7; 8)
Retract pelvic limb ^f^	GA	0 (0; 0)	0 (0; 0)	0 (−0.2; 0)	0 (0; 0)	0 (0; 0.2)	0 (−0.5; 0.2)	0 ^B^ (0; 0)
	GAA	0 (0; 0)	0 (0; 0)	0 (0; 0)	0 (0; 0)	0 (0; 0)	0 (0; 0)	0 ^B^ (0; 0)
	GC	0 (0; 0)	0 (0; 3.7)	3 (−0.5; 12)	1.5 (0; 10.5)	0 (0; 13.2)	0 (0; 0)	1 ^A^ (0; 2)
	GCA	0 (0; 0)	0 (0; 0)	0 (0; 1.2)	0 (0; 0.7)	0 (0; 0)	0 (0; 0.2)	0 ^B^ (0; 0.2)
Expose the penis ^f^	GA	0 ^A^ (0; 0)	0 (0; 0)	0 (−0.2; 0)	0 (0; 0.2)	0 (−0.7; 0.5)	0 (0; 0.5)	0 (0; 0)
	GAA	0 ^A^ (0; 0.2)	0 (−2; 0)	0 (−1; 0)	0 (−0.5; 0)	0 (0; 0)	0 (−2.2; 0)	0 (0; 0.7)
	GC	−3 ^B^ (−3; −1.5)	−2.5 (−4; 0)	0 (0; 0.5)	−1 (−3.5; 0.5)	0 (−0.7; 4.5)	0 (−1.2; 0)	0 (−3; 2)
	GCA	0 ^A^ (−0.5; 0.5)	−1 (−2; 2.2)	−0.5 (−3; 3.7)	0 (0; 6)	−1 (−9.5; 1.2)	−2 (−2.2; 0.2)	0 (−1; 3)

**Table 3:** Median and interquartile range (Q1; Q3) of the behavior duration of 24 horses recorded 24 h before anesthesia for all groups together. Different lowercase letters indicate significant difference over time (a > b > c). AR = after the end of the anesthetic recovery (to investigate the effect of the recording time, the frequency, and duration of behaviors were analyzed on the day before anesthesia combining all groups, because all horses were under the same conditions and had not undergone any procedure yet).

Behaviors	Behavioral Recording Time-Points before Anesthesia					
	−24 h (≈1 h AR)	−22 h (≈2 h AR)	−20 h (≈4 h AR)	−18 h (≈6 h AR)	−16 h (≈8 h AR)	−12 h (≈12 h AR)
	09:54 ± 00:46	11:13 ± 00:24	13:07 ± 00:30	15:06 ± 00:26	17:06 ± 00:29	20:57 ± 00:42
Walk	1 ^ab^ (0.25–2)	1 ^ab^ (0–1)	1 ^ab^ (0.25–3)	1 ^ab^ (1–2)	2 ^a^ (1–4)	0.5 ^b^ (0–1.75)
Rest standing still	0 ^ab^ (0–25.5)	0 ^b^ (0–13)	3 ^b^ (0–16.5)	11 ^ab^ (0–16.75)	2.5 ^b^ (0–11)	24 ^a^ (7–42.25)
Look out the window	9.5 ^a^ (3.25–22)	10.5 ^a^ (2–21)	13.5 ^a^ (5–38.5)	15.5 ^a^ (1.5–26)	8 ^a^ (1–23.25)	0 ^b^ (0–3.75)
Rest the pelvic limb	12 ^ab^ (0.25–21)	3 ^b^ (0–11.75)	7.5 ^ab^ (2–22.75)	12 ^ab^ (3–22.75)	6 ^b^ (1–11)	10 ^a^ (2–39)

**Table 4:** Summary of the changes in equine behaviors influenced by time of day *, not affected by analgesia or anesthesia and related only with pain in this study ** and described in the literature in different equine pain conditions [1,2,3,4,5,6,7,8,9,10,11,12,13,14,15,16,17,18,19,20,21,22,23,24,25,36,41]. The delta (∆) of behaviors that proportionally increased (↑), decreased (↓), or both (↑↓) exclusively in the GC compared to the other groups in at least one time-point and the behaviors for which the duration or frequency were affected by the time of the day (before anesthesia). The delta (∆) is the subtraction of the frequency or duration of the behaviors recorded after the anesthesia from those recorded in the mirrored hours before the anesthesia. GC = horses submitted to anesthesia and orchiectomy with postoperative analgesia; ^d^ indicates behaviors assessed as duration and ^f^ as frequency.

Behaviors	Behavior Influenced by Time of Day (before Anesthesia-all Groups) *	Pain		
		Behavior Specifically Related to Pain in this Study (GC) **	Pain Behavior according to Literature	Neither Described by Literature nor Present in this Study
Drink ^d^		↓	X	
Eat ^d^		↓	X	
Swing tail			X	
Kick			X	
Retract pelvic limb ^f^		↑	X	
Retract and extend pelvic limb			X	
Look at the wound ^f^		↑	X	
Smell				X
Flehmen			X	
Look at the back of the stall ^d^		↓	X	
Look out the window ^d^	↓↑		X	
Stare at the side of the stall			X	
Stay at the back of the stall			X	
Scratch			X	
Lower head			X	
Head below withers			X	
Rest standing still ^d^	↓↑		X	
Rest pelvic limb ^d^	↓↑		X	
Try to lie down			X	
Yawn			X	
Raise the tail				X
Paw			X	
Sternal decubitus			X	
Lateral decubitus			X	
Movements with the tongue			X	
Vertical movement of the head			X	
Masticatory movements				X
Roll			X	
Defecate			X	
Urinate			X	
Walk ^d^	↓↑	↓	X	
Expose the penis ^f^		↓		
Stretch the body			X	
Shake			X	

## Data Availability

The data presented in this study are available in the supplementary material according to “MDPI Research Data Policies” at https://www.mdpi.com/ethics.

## Supplementary Materials

The following are available online at https://www.mdpi.com/article/10.3390/ani11061629/s1. Table S1: Raw data median and interquartile range (Q_1_; Q_3_) of the frequency (^f^) or duration (^d^) of the behaviors that showed differences in delta (Table 2) between groups of horses submitted to anesthesia (GA), preoperative analgesia and anesthesia (GAA), anesthesia, orchiectomy and postoperative analgesia (GC) and, anesthesia, preoperative analgesia and orchiectomy (GCA). BR = before the end of anesthesia recovery; AR = after the anesthesia recovery and Table S2: Raw data median and interquartile range (Q1; Q3) of the frequency (^f^) or duration (^d^) of the six pain-related behaviors unaffected by the time of day, anesthesia, analgesia and surgery. Horses were submitted to anesthesia (GA), previous analgesia and anesthesia (GAA), preoperative analgesia, anesthesia and orchiectomy (GCA) and anesthesia, orchiectomy and postoperative analgesia (GC). Different lowercase letters and values in bold indicate a significant difference over time in each group (a > b > c), and different uppercase letters and values in bold indicate a significant difference between groups at each time-point (A > B > C).

## References

[1] Andersen P.H., Gleerup K.B., Wathan J., Coles B., Kjellström H., Broomé S., Lee Y.J., Rashid M., Sonder C., Resenberg E. Can a machine learn to see horse pain? An interdisciplinary approach towards automated decoding of facial expressions of pain in the horse. *Measuring Behavior 2018-11th International Conference on Methods and Techniques in Behavioral Research*, Proceedings of Measuring Behavior, Manchester, UK, 6–8 June 2018. www.measuringbehavior.org.

[2] Guedes A. (2017). Pain management in horses. Vet. Clin. North. Am. Equine Pract..

[3] Dugdale A.H.A. (2014). Progress in equine pain assessment?. Vet. J..

[4] Wagner A.E. (2010). Effects of stress on pain in horses and incorporating pain scales for equine practice. Vet. Clin. North. Am. Equine Pract..

[5] Sutton G.A., Dahan R., Turner D., Paltiel O. (2013). A behaviour-based pain scale for horses with acute colic: Scale construction. Vet. J..

[6] Sutton G.A., Atamna R., Steinman A., Mair T.S. (2019). Comparison of three acute colic pain scales: Reliability, validity and usability. Vet. J..

[7] VanDierendonck M.C., van Loon J.P.A.M. (2016). Monitoring acute equine visceral pain with the Equine Utrecht University Scale for Composite Pain Assessment (EQUUS-COMPASS) and the Equine Utrecht University Scale for Facial Assessment of Pain (EQUUS-FAP): A validation study. Vet. J..

[8] Sutton G.A., Paltiel O., Soffer M., Turner D. (2013). Validation of two behaviour-based pain scales for horses with acute colic. Vet. J..

[9] Sutton G.A., Sutton G.A., Bar L., Sutton G. (2016). Evaluation of pain in horses refinement and revalidation of the Equine Acute Abdominal Pain Scale (EAAPS). Isr J. Vet. Med..

[10] Graubner C., Gerber V., Doherr M., Spadavecchia C. (2011). Clinical application and reliability of a post abdominal surgery pain assessment scale (PASPAS) in horses. Vet. J..

[11] Pritchett L.C., Ulibarri C., Roberts M.C., Schneider R.K., Sellon D.C. (2003). Identification of potential physiological and behavioral indicators of postoperative pain in horses after exploratory celiotomy for colic. Appl. Anim. Behav. Sci..

[12] van Loon J.P.A.M., Van Dierendonck M.C. (2015). Monitoring acute equine visceral pain with the Equine Utrecht University Scale for Composite Pain Assessment (EQUUS-COMPASS) and the Equine Utrecht University Scale for Facial Assessment of Pain (EQUUS-FAP): A scale-construction study. Vet. J..

[13] van Loon J.P.A.M., Back W., Hellebrekers L.J., van Weeren P.R. (2010). Application of a composite pain scale to objectively monitor horses with somatic and visceral pain under hospital conditions. J. Equine Vet. Sci..

[14] Sellon D.C., Roberts M.C., Blikslager A.T., Ulibarri C., Papich M.G. (2004). Effects of continuous rate intravenous infusion of butorphanol on physiologic and outcome variables in horses after celiotomy. J. Vet. Intern. Med..

[15] Van Loon J.P.A.M., Jonckheer-Sheehy V.S.M., Back W., René van Weeren P., Hellebrekers L.J. (2014). Monitoring equine visceral pain with a composite pain scale score and correlation with survival after emergency gastrointestinal surgery. Vet. J..

[16] Lawson A.L., Opie R.R., Stevens K.B., Knowles E.J., Mair T.S. (2020). Application of an equine composite pain scale and its association with plasma adrenocorticotropic hormone concentrations and serum cortisol concentrations in horses with colic. Equine Vet. Educ..

[17] Pehkonen J., Karma L., Raekallio M. (2019). Behavioral signs associated with equine periapical infection in cheek teeth. J. Equine Vet. Sci..

[18] Taffarel M.O., Luna S.P.L., de Oliveira F.A., Cardoso G.S., de Moura Alonso J., Pantoja J.C., Brondani J.T., Love E., Taylor P., White K. (2015). Refinement and partial validation of the UNESP-Botucatu multidimensional composite pain scale for assessing postoperative pain in horses. BMC Vet. Res..

[19] Dalla Costa E., Minero M., Lebelt D., Stucke D., Canali E., Leach M.C. (2014). Development of the Horse Grimace Scale (HGS) as a pain assessment tool in horses undergoing routine castration. PLoS ONE.

[20] Price J., Catriona S., Welsh E.M., Waran N.K. (2003). Preliminary evaluation of a behaviour-based system for assessment of post-operative pain in horses following arthroscopic surgery. Vet. Anaesth Analg..

[21] van Loon J.P.A.M., Van Dierendonck M.C. (2019). Pain assessment in horses after orthopaedic surgery and with orthopaedic trauma. Vet. J..

[22] Bussières G., Jacques C., Lainay O., Beauchamp G., Leblond A., Cadoré J.-L., Desmaizières M., Cuvelliez S.G., Troncy E. (2008). Development of a composite orthopaedic pain scale in horses. Res. Vet. Sci..

[23] Lindegaard C., Thomsen M.H., Larsen S., Andersen P.H. (2010). Analgesic efficacy of intra-articular morphine in experimentally induced radiocarpal synovitis in horses. Vet. Anaesth Analg..

[24] Dutton D.W., Lashnits K.J., Wegner K. (2009). Managing severe hoof pain in a horse using multimodal analgesia and a modified composite pain score. Equine Vet. Educ..

[25] Raekallio M., Taylor P.M., Bloomfield M. (1997). A comparison of methods for evaluation of pain and distress after orthopaedic surgery in horses. J. Vet. Anaesth..

[26] Gleerup K.B., Lindegaard C. (2016). Recognition and quantification of pain in horses: A tutorial review. Equine Vet. Educ..

[27] Torcivia C., McDonnell S. (2020). In-person caretaker visits disrupt ongoing discomfort behavior in hospitalized equine orthopedic surgical patients. Animals.

[28] Gleerup K.B., Forkman B., Lindegaard C., Andersen P.H. (2015). An equine pain face. Vet. Anaesth Analg..

[29] Luna S.P.L., de Araújo A.L., da Nóbrega Neto P.I., Brondani J.T., de Oliveira F.A., Azerêdo LM dos S., Telles F.G., Trindade P.H.E. (2020). Validation of the UNESP-Botucatu pig composite acute pain scale (UPAPS). PLoS ONE.

[30] Silva N.E.O.F., Trindade P.H.E., Oliveira A.R., Taffarel M.O., Moreira M.A.P., Denadai R., Rocha P.B., Luna S.P.L. (2020). Validation of the Unesp-Botucatu composite scale to assess acute postoperative abdominal pain in sheep (USAPS). PLoS ONE.

[31] Descovich K.A., Wathan J., Leach M.C., Buchanan-Smith H.M., Flecknell P., Farningham D., Vick S.J. (2017). Facial expression: An under-utilized tool for the assessment of welfare in mammals. Altex.

[32] Mcdonnell S.M. Is it psychological, physical, or both?. Proceedings of the 51st Annual Convention of the American Association of Equine Practitioners.

[33] Dodds L., Knight L., Allen K., Murrell J. (2017). The effect of postsurgical pain on attentional processing in horses. Vet. Anaesth Analg..

[34] Hall C., Randle H., Pearson G., Preshaw L., Waran N. (2018). Assessing equine emotional state. Appl. Anim. Behav. Sci..

[35] Gigliuto C., De Gregori M., Malafoglia V., Raffaeli W., Compagnone C., Visai L., Petrini P., Avanzini M.A., Muscoli C., Viganò J. (2014). Pain assessment in animal models: Do we need further studies?. J. Pain Res..

[36] Ashley F.H., Waterman-Pearson A.E., Whay H.R. (2005). Behavioural assessment of pain in horses and donkeys: Application to clinical practice and future studies. Equine Vet. J..

[37] Pinho R.H., Leach M.C., Minto B.W., Rocha F.D.L., Luna S.P.L. (2020). Postoperative pain behaviours in rabbits following orthopaedic surgery and effect of observer presence. PLoS ONE.

[38] McDonnell S. (2003). The Equid Ethogram: A Practical Field Guide to Horse Behaviour.

[39] Taffarel M.O., Luna S.P.L., Cardoso G.S., de Oliveira F.A., de Moura Alonso J., Gozalo-Marcilla M. (2017). Preemptive Analgesia, including morphine, does not affect recovery quality and times in either pain-free horses or horses undergoing orchiectomy. J. Equine Vet. Sci..

[40] Searle D., Dart A.J., Dart C.M., Hodgson D.R. (1999). Equine castration: Review of anatomy, approaches, techniques and complications in normal, cryptorchid and monorchid horses. Aust Vet. J..

[41] Torcivia C., Mcdonnell S. (2020). Equine discomfort ethogram. Animals.

[42] Martin P., Bateson P.P.G. (1993). Measuring Behaviour: An Introductory Guide.

[43] Steagall P., Luna S., Taylor P., Humm K.F.T. (2009). Neurological, respiratory, bahavioural and endocrine effects of tail docking in newborn dogs submitted to epidural anesthesia. Ars Vet..

[44] Valbrun L.P., Zvonarev V. (2020). The opioid system and food intake: Use of opiate antagonists in treatment of binge eating disorder and abnormal eating behavior. J. Clin. Med. Res..

[45] Bailey P.A., Hague B.A., Davis M., Major M.D., Zubrod C.J., Brakenhoff J.E. (2016). Incidence of post-anesthetic colic in non-fasted adult equine patients. Can. Vet. J..

[46] Brondani J.T., Mama K.R., Luna S.P.L., Wright B.D., Niyom S., Ambrosio J., Vogel P.R., Padovani C.R. (2013). Validation of the English version of the UNESP-Botucatu multidimensional composite pain scale for assessing postoperative pain in cats. BMC Vet. Res..

[47] de Oliveira F.A., Luna S.P.L., do Amaral J.B., Rodrigues K.A., Sant’Anna A.C., Daolio M., Brondani J.T. (2014). Validation of the UNESP-Botucatu unidimensional composite pain scale for assessing postoperative pain in cattle. BMC Vet. Res..

[48] Streiner D.L., Norman G.R., Cairney J. (2015). Health Measurement Scales—A Practical Guide to Their Development and Use.

[49] Devine E.P., Kukanich B., Beard W.L. (2013). Pharmacokinetics of intramuscularly administered morphine in horses. J. Am. Vet. Med. Assoc..

[50] Klaus A., Schlingloff Y., Kleinitz U., Bottcher M., Hapke H.J. (1997). Pharmacokinetic study of dipyrone metabolite 4-MAA in the horse and possible implications for doping control. J. Vet. Pharmacol Ther..

[51] Toutain P.L., Autefage A., Legrand C., Alvinerie M. (1994). Plasma concentrations and therapeutic efficacy of phenylbutazone and flunixin meglumine in the horse: Pharmacokinetic/pharmacodynamic modelling. J. Vet. Pharmacol Ther..

[52] Leach M.C., Allweiler S., Richardson C., Roughan J.V., Narbe R., Flecknell P.A. (2009). Behavioural effects of ovariohysterectomy and oral administration of meloxicam in laboratory housed rabbits. Res. Vet. Sci..

[53] Duncan P. (1983). Determinants of the use of habitat by horses in a Mediterranean Wetland. J Anim Ecol..

[54] Melgaard D.T., Korsgaard T.S., Thoefner M.S., Petersen M.R., Pedersen H.G. (2020). Moody mares—Is ovariectomy a solution?. Animals.

[55] Bulens A., Van Beirendonck S., Van Thielen J., Driessen B. (2013). The enriching effect of non-commercial items in stabled horses. Appl. Anim. Behav. Sci..

[56] Forkman B., Boissy A., Meunier-Salaün M.C., Canali E., Jones R.B. (2007). A critical review of fear tests used on cattle, pigs, sheep, poultry and horses. Physiol. Behav..

