# Supplementary material for: Spontaneous Behaviors of Post-Orchiectomy Pain in Horses Regardless of the Effects of Time of Day, Anesthesia, and Analgesia
Source: Animals (Basel). 2021 May 31;11(6):1629. doi: 10.3390/ani11061629 (PMC8230028; doi:10.3390/ani11061629)
Supplement: Supplementary file 1 [file animals-11-01629-s001.zip › Table S1 25.05.21.pdf]

Table S1. Raw data median and interquartile range (Q_1; Q_3) of the frequency (^{f}) or duration (^{d}) of the behaviors that showed differences in delta (Table 2) between groups of horses submitted to anesthesia (GA), preoperative analgesia and anesthesia (GAA), anesthesia, orchiectomy and postoperative analgesia (GC) and, anesthesia, preoperative analgesia and orchiectomy (GCA). BR = before the end of anesthesia recovery; AR = after the anesthesia recovery.

			Time-points						
**Behaviors**	**Groups**		**1 h AR and 24hBR**	**2hAR and 22hBR**	**4hAR and 20hBR**	**6hAR and 18hBR**	**8hAR and 16hBR**	**12hAR and 12hBR**	**24hAR and 24hBR**
**Drink^{d}**	**GA**	**BR**	0 (0; 1)	0 (0; 1)	0 (0; 0.25)	0 (0; 1)	0 (0; 0.25)	0 (0; 1)	0 (0; 1)
		**AR**	0 (0; 0.25)	0 (0; 1)	0.5 (0; 1.25)	0 (0; 1)	0 (0; 1)	0 (0; 0.5)	0 (0; 1)
	**GAA**	**BR**	0 (0; 0)	1 (0.75; 1)	0.5 (0; 1)	0 (0; 0)	0 (0; 0.25)	0 (0; 0.25)	0 (0; 0)
		**AR**	0 (0; 0.25)	0 (0; 0)	0 (0; 1)	1 (0; 1)	0.5 (0; 1)	0 (0; 0.25)	0 (0; 1.25)
	**GC**	**BR**	0.5 (0; 1.25)	0.5 (0; 2.5)	1 (0; 1)	1 (0; 1)	0 (0; 1.25)	1 (0; 1.5)	0.5 (0; 1.25)
		**AR**	0 (0; 0)	0 (0; 0)	0 (0; 0.25)	0 (0; 0)	0 (0; 0.5)	0.5 (0; 1)	1 (0.75; 1.75)
	**GCA**	**BR**	0 (0; 0)	0.5 (0; 1)	0 (0; 0.25)	0.5 (0; 1)	0 (0; 0)	0 (0; 0.25)	0 (0; 0)
		**AR**	0 (0; 0)	0 (0; 0)	0 (0; 1)	0 (0; 1)	0 (0; 1)	0 (0; 0.25)	0 (0; 0.25)
**Eat^{d}**	**GA**	**BR**	33.5 (8.25; 45.5)	30 (15.75; 39.75)	16 (8.25; 29.75)	30 (17.25; 41.75)	22 (16; 35.75)	22.5 (15.75; 43.25)	33.5 (8.25; 45.5)
		**AR**	30 (28.75; 36.75)	34.5 (25.75; 48.5)	35 (24.75; 38.75)	32 (18.5; 45.5)	23 (9; 33)	34.5 (11.75; 46.25)	28 (17.75; 53.75)
	**GAA**	**BR**	25.5 (13.5; 38.75)	39 (15; 45.75)	19.5 (9.5; 30)	22.5 (11.5; 45.25)	19.5 (9.75; 46.75)	18.5 (3; 39)	25.5 (13.5; 38.75)
		**AR**	25 (17.25; 30.5)	30.5 (17.25; 38)	24.5 (13.75; 47.25)	30.5 (19; 48.75)	33.5 (25.75; 47)	15.5 (9.75; 22.5)	15 (5.25; 34.5)
	**GC**	**BR**	25 (12.5; 38)	28.5 (10.5; 46)	45 (31.75; 51.25)	34 (24.25; 45.75)	33.5 (20.5; 41.75)	41.5 (9; 51.5)	25 (12.5; 38)
		**AR**	4.5 (0.75; 21.25)	2 (0; 21.5)	10 (2.25; 24.75)	51.5 (29.25; 57.25)	47 (44.5; 54)	42.5 (25.75; 48.25)	31.5 (18.75; 39.25)
	**GCA**	**BR**	32.5 (21.5; 43.75)	13.5 (7.5; 33.75)	28 (19.75; 62)	39.5 (24.75; 46)	38.5 (27.5; 46.75)	24 (7; 43)	32.5 (21.5; 43.75)
		**AR**	29 (22.75; 34.25)	34 (23.25; 45)	46.5 (29.5; 60.25)	34.5 (23.75; 52.5)	24 (3.75; 48.75)	26.5 (9.75; 38.5)	28.5 (18; 33.5)
**Defecate^{f}**	**GA**	**BR**	1 (0; 1)	1 (0; 1)	1 (0.75; 1.25)	1 (0; 2)	1 (0; 2.75)	1 (0; 1.25)	1 (0; 1)
		**AR**	0.5 (0; 1.5)	1 (0.75; 2)	1 (1; 2.25)	1 (1; 2.25)	0.5 (0; 2.5)	1 (0; 2)	1 (0; 1.5)
	**GAA**	**BR**	1 (0; 1.25)	0.5 (0; 1)	1.5 (1; 2)	1 (0; 1)	2 (0; 4)	0 (0; 0)	1 (0; 1.25)
		**AR**	0 (0; 0)	0 (0; 0.75)	0.5 (0; 1.25)	0.5 (0; 1.5)	1 (0.75; 2.25)	0.5 (0; 1.25)	1 (0.75; 1.25)
	**GC**	**BR**	0.5 (0; 1)	1 (0; 1)	1 (0; 1.25)	0.5 (0; 1)	1 (0.75; 1.5)	1 (0; 1.25)	0.5 (0; 1)
		**AR**	0.5 (0; 1.25)	0 (0; 0.25)	0 (0; 0.25)	0 (0; 0.25)	0 (0; 0.25)	1 (0; 1.25)	1 (0.75; 1)
	**GCA**	**BR**	1 (0.75; 1.25)	1 (0.75; 1.25)	1 (0.75; 1)	1 (0; 1)	1 (1; 1.25)	1 (0.75; 1)	1 (0.75; 1.25)
		**AR**	0 (0; 0.75)	0 (0; 0.25)	1 (0; 1.25)	0.5 (0; 1.25)	1 (0; 2)	1 (0; 1)	1 (0; 1.25)
**Walk^{d}**	**GA**	**BR**	2 (0.75; 3.5)	1 (0.75; 3)	2 (0.75; 6)	1 (1; 2.75)	4.5 (1.75; 6.25)	0.5 (0; 2.75)	2 (0.75; 3.5)
		**AR**	1 (0; 2.5)	1.5 (0.75; 5)	4 (1; 8)	5 (2.5; 7.25)	2 (0.75; 5)	0.5 (0; 1.25)	3.5 (1; 6.75)
	**GAA**	**BR**	0.5 (0; 3)	0.5 (0; 1.5)	0.5 (0; 3)	1.5 (0; 3)	2.5 (0; 4.25)	0 (0; 1.25)	0.5 (0; 3)
		**AR**	0 (0; 2.25)	0.5 (0; 6.5)	3.5 (1; 9)	3.5 (1; 6.25)	3 (0.75; 6)	1 (0.75; 2.25)	4 (0.75; 10.75)
	**GC**	**BR**	1 (1; 1.25)	1 (0.75; 1.25)	1 (0.75; 1.5)	1 (1; 2)	1 (1; 2.5)	0.5 (0; 1.25)	1 (1; 1.25)
		**AR**	1 (0.75; 2.25)	1.5 (0.75; 3.25)	1 (0.75; 1.25)	0 (0; 0)	1 (0; 2)	1 (1; 1.5)	1 (1; 1.75)
	**GCA**	**BR**	1 (0; 3.5)	1 (0; 2)	1 (0.75; 2.5)	1 (0; 1.25)	0.5 (0; 3.5)	1 (0; 2.5)	1 (0; 3.5)
		**AR**	0 (0; 1.75)	1 (0; 7)	1 (0; 3.75)	1.5 (0.75; 5)	1.5 (0; 4.25)	2 (0.75; 2.25)	1.5 (0.75; 2.5)

**Rest standing still**[d]	**GA**	**BR**	0 (0; 0.5)	0 (0; 3.75)	0 (0; 8.75)	0 (0; 13)	3 (0; 10.75)	24.5 (11.5; 40.75)	0 (0; 0.5)
		**AR**	0 (0; 0)	0 (0; 0.25)	3.5 (0; 6.75)	5 (0; 6.75)	2.5 (0; 12)	11.5 (0.75; 42.5)	0 (0; 0.25)
	**GAA**	**BR**	0 (0; 12.75)	0 (0; 9.5)	10 (0; 23.5)	11 (0; 19.75)	0 (0; 5)	35.5 (19.25; 57)	0 (0; 12.75)
		**AR**	0 (0; 0)	0 (0; 2.75)	1.5 (0; 4)	1.5 (0; 4.75)	0 (0; 6.25)	31 (21.5; 41.5)	3.5 (0.75; 8.25)
	**GC**	**BR**	28.5 (15.75; 37.5)	11.5 (0.75; 18.25)	8 (0; 13.25)	17 (10.5; 22)	12 (3; 15.5)	15 (3.75; 46.5)	28.5 (15.75; 37.5)
		**AR**	10 (3.75; 21.5)	17.5 (3.25; 28.75)	29.5 (20.5; 40.75)	1.5 (0; 15.75)	0 (0; 5)	11 (8.75; 26)	24.5 (4.5; 35.25)
	**GCA**	**BR**	8 (0; 22.25)	4.5 (0; 16)	5.5 (0; 12.75)	5.5 (0; 14)	0 (0; 7.5)	14 (4.5; 38.25)	8 (0; 22.25)
		**AR**	0 (0; 0)	0 (0; 0.25)	0 (0; 0.25)	3 (0; 20.25)	2 (0; 4.25)	31.5 (7; 41)	13.5 (0; 27.5)
**Stay at the back of the stall**[d]	**GA**	**BR**	0 (0; 0)	0 (0; 0)	0 (0; 0.5)	0 (0; 0)	0 (0; 2.75)	0 (0; 0)	0 (0; 0)
		**AR**	0 (0; 0)	0 (0; 0)	0 (0; 0)	0 (0; 1.75)	0 (0; 3.5)	0 (0; 0.25)	0 (0; 0)
	**GAA**	**BR**	0 (0; 0)	0 (0; 0.25)	0 (0; 0)	0 (0; 0)	0 (0; 0.5)	0 (0; 0)	0 (0; 0)
		**AR**	0 (0; 0)	0 (0; 0.5)	0 (0; 0)	0 (0; 0)	0 (0; 0)	0 (0; 0)	0 (0; 0.25)
	**GC**	**BR**	0 (0; 20.5)	0 (0; 5.5)	0 (0; 10)	2 (0; 7.5)	0 (0; 3.5)	0 (0; 8)	0 (0; 20.5)
		**AR**	0 (0; 0)	0 (0; 4.5)	0 (0; 5)	0 (0; 0)	0 (0; 4.5)	0 (0; 9.75)	3.5 (0; 25)
	**GCA**	**BR**	0 (0; 9.25)	0 (0; 1.75)	0 (0; 3.5)	0 (0; 9.25)	0 (0; 0)	0 (0; 1.75)	0 (0; 9.25)
		**AR**	0 (0; 0)	0 (0; 0)	0 (0; 0)	0 (0; 0)	0 (0; 0.25)	1 (0; 7.25)	0 (0; 5.5)
**Look out the window**[d]	**GA**	**BR**	23 (10.25; 49)	18.5 (9; 33.25)	32.5 (12; 40.5)	25 (13.25; 34)	16 (8.5; 25.5)	4.5 (0.75; 9)	23 (10.25; 49)
		**AR**	3.5 (0.75; 5)	4 (1; 12.75)	21 (15.5; 37.25)	16 (9; 31.75)	20.5 (8.5; 32.75)	5 (2.25; 10.5)	26.5 (3.75; 38)
	**GAA**	**BR**	21 (16; 37.5)	8 (3; 19.5)	24 (7; 51)	22 (12; 30)	15 (5.5; 34.25)	0 (0; 3.5)	21 (16; 37.5)
		**AR**	0 (0; 2.75)	0 (0; 4.5)	24 (5.75; 41)	20 (2.75; 27)	10.5 (6.25; 18.5)	10.5 (2.25; 12.75)	31 (9; 40)
	**GC**	**BR**	3.5 (2.25; 4.25)	0.5 (0; 5)	5 (0; 11.25)	2 (0; 12.25)	1 (0.75; 11)	0 (0; 2.5)	3.5 (2.25; 4.25)
		**AR**	4.5 (0; 7)	3 (1.5; 9)	6 (0.75; 13.25)	0.5 (0; 3)	0.5 (0; 3)	0.5 (0; 5)	5 (1.75; 10.25)
	**GCA**	**BR**	7.5 (0; 11.75)	19 (2.75; 26)	19 (1.5; 34.75)	4 (0.75; 19.75)	3.5 (0; 24)	0 (0; 3)	7.5 (0; 11.75)
		**AR**	0 (0; 1.75)	0 (0; 1.5)	7 (0; 21)	8.5 (1.5; 24.25)	11.5 (5.25; 22)	2.5 (1.5; 14.75)	12.5 (3.75; 18)
**Look at the back of the stall**[d]	**GA**	**BR**	0 (0; 0)	0 (0; 0)	0 (0; 0)	0 (0; 0)	0 (0; 1.75)	0 (0; 10.75)	0 (0; 0)
		**AR**	0 (0; 0)	0 (0; 0)	0 (0; 0)	0 (0; 0.25)	0 (0; 0)	0 (0; 0.25)	0 (0; 0)
	**GAA**	**BR**	0 (0; 0)	0 (0; 0.25)	0 (0; 0)	0 (0; 0.5)	0 (0; 0.25)	0 (0; 0)	0 (0; 0)
		**AR**	0 (0; 0)	0 (0; 3)	0 (0; 0)	0 (0; 2.25)	0 (0; 0)	0 (0; 0)	0.5 (0; 2.25)
	**GC**	**BR**	13 (1.5; 31.5)	0 (0; 5.5)	0 (0; 3)	0.5 (0; 16.5)	0 (0; 12.5)	0 (0; 12.5)	13 (1.5; 31.5)
		**AR**	0 (0; 2.5)	1.5 (0; 18.5)	1 (0; 19)	0 (0; 1)	0 (0; 0.75)	2.5 (0; 22.25)	2 (0; 3.5)
	**GCA**	**BR**	0.5 (0; 5.5)	0 (0; 0)	0 (0; 1.25)	0 (0; 1.25)	0 (0; 0)	0 (0; 0.75)	0.5 (0; 5.5)
		**AR**	0 (0; 0)	0 (0; 0)	0 (0; 0)	0 (0; 0.25)	0 (0; 1)	0 (0; 1.5)	0 (0; 0.25)
**Look at the wound**[f]	**GA**	**BR**	0 (0; 0)	0 (0; 0)	0 (0; 0.5)	0 (0; 0.5)	0 (0; 0)	0 (0; 0)	0 (0; 0)
		**AR**	0 (0; 0)	0 (0; 0)	0 (0; 0)	0 (0; 0)	0 (0; 0)	0 (0; 1.25)	0 (0; 0)
	**GAA**	**BR**	0 (0; 0)	0 (0; 0)	0 (0; 0)	0 (0; 0)	0 (0; 0)	0 (0; 0)	0 (0; 0)
		**AR**	0 (0; 0)	0 (0; 0)	0 (0; 0)	0 (0; 0)	0 (0; 0)	0 (0; 0)	0 (0; 0)
	**GC**	**BR**	0 (0; 0.5)	0 (0; 0)	0 (0; 0)	0 (0; 0)	0 (0; 0)	0 (0; 0)	0 (0; 0.5)
		**AR**	0 (0; 4.25)	1 (0; 7.75)	0 (0; 3.5)	0 (0; 2.75)	0 (0; 1.25)	0 (0; 0.5)	0 (0; 0)

	**GCA**	**BR**	0 (0; 0)	0 (0; 0)	0 (0; 0)	0 (0; 0)	0 (0; 0)	0 (0; 0)	0 (0; 0)
		**AR**	0 (0; 0)	0 (0; 0)	0 (0; 2.75)	0 (0; 0.5)	0 (0; 0)	0 (0; 0)	0 (0; 0)
**Rest pelvic limb[d]**	**GA**	**BR**	12 (4.75; 20.5)	7 (3; 19.25)	11 (3.5; 22)	4.5 (3.75; 24)	3.5 (1; 14)	22.5 (7.75; 36)	12 (4.75; 20.5)
		**AR**	1 (0; 4.5)	2.5 (0.75; 6)	16 (8.25; 19.5)	8.5 (2.75; 13.5)	12 (7.25; 15.5)	23 (4.75; 47.5)	3 (0; 9.5)
	**GAA**	**BR**	18.5 (0; 35)	8 (0; 19.5)	22.5 (14.5; 32)	21 (15.5; 28.75)	6.5 (0; 14.25)	36 (1.5; 53.25)	18.5 (0; 35)
		**AR**	0 (0; 0.25)	0 (0; 2.5)	10.5 (0; 30.25)	1 (0; 14.25)	9 (0; 14.5)	33.5 (20.25; 43)	7.5 (0.75; 14.25)
	**GC**	**BR**	15.5 (1.5; 22.75)	1 (0; 9)	3.5 (1.75; 10.25)	11 (2.5; 23.5)	8 (0.75; 11.25)	9.5 (3.75; 46.25)	15.5 (1.5; 22.75)
		**AR**	10.5 (1.5; 22.5)	15 (0; 27)	39.5 (6.5; 48)	2.5 (0; 24.75)	0 (0; 2.5)	9.5 (0.75; 15.75)	0 (0; 8.75)
	**GCA**	**BR**	0.5 (0; 15)	0.5 (0; 7.25)	3 (0; 26)	1.5 (0; 13)	7.5 (0; 12)	1.5 (0.75; 16)	0.5 (0; 15)
		**AR**	0 (0; 0)	0 (0; 5.25)	0.5 (0; 4.25)	2.5 (0; 8.25)	4.5 (0.75; 9.75)	10.5 (0; 23)	7 (0; 12.75)
**Retract pelvic limb[f]**	**GA**	**BR**	0 (0; 0)	0 (0; 0)	0 (0; 0.25)	0 (0; 0)	0 (0; 0)	0 (0; 0.5)	0 (0; 0)
		**AR**	0 (0; 0)	0 (0; 0)	0 (0; 0)	0 (0; 0)	0 (0; 0.25)	0 (0; 0.25)	0 (0; 0)
	**GAA**	**BR**	0 (0; 0)	0 (0; 0)	0 (0; 0)	0 (0; 0)	0 (0; 0)	0 (0; 0)	0 (0; 0)
		**AR**	0 (0; 0)	0 (0; 0)	0 (0; 0)	0 (0; 0)	0 (0; 0)	0 (0; 0)	0 (0; 0)
	**GC**	**BR**	0 (0; 0)	0 (0; 0.25)	0 (0; 0.5)	0 (0; 0.5)	0 (0; 0)	0 (0; 0)	0 (0; 0)
		**AR**	0 (0; 0)	0 (0; 4)	3 (0; 12)	1.5 (0; 12)	0 (0; 13.25)	0 (0; 0)	1 (0; 2)
	**GCA**	**BR**	0 (0; 0)	0 (0; 0)	0 (0; 0)	0 (0; 0)	0 (0; 0)	0 (0; 0)	0 (0; 0)
		**AR**	0 (0; 0)	0 (0; 0)	0 (0; 1.25)	0 (0; 0.75)	0 (0; 0)	0 (0; 0.25)	0 (0; 0.25)
**Expose the penis[f]**	**GA**	**BR**	0 (0; 0)	0 (0; 0)	0 (0; 0.25)	0 (0; 0)	0 (0; 0.75)	0 (0; 0.25)	0 (0; 0)
		**AR**	0 (0; 0)	0 (0; 0)	0 (0; 0)	0 (0; 0.25)	0 (0; 0.5)	0 (0; 0.75)	0 (0; 0)
	**GAA**	**BR**	0 (0; 0)	0 (0; 2.75)	0 (0; 3.25)	0 (0; 0.5)	0 (0; 0)	0 (0; 2.25)	0 (0; 0)
		**AR**	0 (0; 0.25)	0 (0; 0.25)	0 (0; 2.25)	0 (0; 0)	0 (0; 0)	0 (0; 0)	0 (0; 0.75)
	**GC**	**BR**	3 (1.5; 3)	2.5 (0; 4)	0 (0; 0)	2.5 (0; 3.5)	0 (0; 0.75)	0 (0; 2.75)	3 (1.5; 3)
		**AR**	0 (0; 0)	0 (0; 0)	0 (0; 0.5)	0 (0; 1.25)	0 (0; 4.5)	0 (0; 1.5)	1.5 (0; 4.25)
	**GCA**	**BR**	0 (0; 2.5)	1 (0; 2)	2.5 (0; 3.75)	0 (0; 3.75)	3 (0; 9.75)	2 (0; 2.25)	0 (0; 2.5)
		**AR**	0 (0; 1.5)	0 (0; 2.25)	0 (0; 8)	2.5 (0; 10.5)	1.5 (0; 2.75)	0 (0; 0.25)	0.5 (0; 3.75)
