# Supplementary material for: Spontaneous Behaviors of Post-Orchiectomy Pain in Horses Regardless of the Effects of Time of Day, Anesthesia, and Analgesia
Source: Animals (Basel). 2021 May 31;11(6):1629. doi: 10.3390/ani11061629 (PMC8230028; doi:10.3390/ani11061629)
Supplement: Supplementary file 1 [file animals-11-01629-s001.zip › Table S2 25.05.21.pdf]

Table S2. Raw data median and interquartile range (Q1; Q3) of the frequency (^{f}) or duration (^{d}) of the six pain-related behaviors unaffected by the time of day, anesthesia, analgesia and surgery. Horses were submitted to anesthesia (GA), previous analgesia and anesthesia (GAA), preoperative analgesia, anesthesia and orchiectomy (GCA) and anesthesia, orchiectomy and postoperative analgesia (GC). Different lowercase letters and values in bold indicate a significant difference over time in each group (a>b>c), and different uppercase letters and values in bold indicate a significant difference between groups at each time-point (A>B>C).

		Time-points							
**Behaviors**	**Groups**	**7 h BR**	**1 h AR**	**2 h AR**	**4 h AR**	**6 h AR**	**8 h AR**	**12 h AR**	**24 h AR**
**Drink^{d}**	**GA**	0 (0; 0)	0 (0; 0.25)	0 (0; 1)	0.5 (0; 1.25)	0 (0; 1)	0 (0; 1)	0 (0; 0.5)	0 (0; 1)
	**GAA**	0 (0; 0)	0 (0; 0.25)	0 (0; 0)	0 (0; 1)	1 (0; 1)	0.5 (0; 1)	0 (0; 0.25)	0 (0; 1.25)
	**GC**	0 (0; 1)	0 (0; 0)	0 (0; 0)	0 (0; 0.25)	0 (0; 0)	0 (0; 0.5)	0.5 (0; 1)	1 (0.75; 1.75)
	**GCA**	0 (0; 0)	0 (0; 0)	0 (0; 0)	0 (0; 1)	0 (0; 1)	0 (0; 1)	0 (0; 0.25)	0 (0; 0.25)
**Eat^{d}**	**GA**	14 (3; 17.75)	**30 ^{A} (28.75; 36.75)**	34.5 (25.75; 48.5)	35 (24.75; 38.75)	32 (18.5; 45.5)	**23 ^{B} (9; 33)**	**34.5 ^{AB} (11.75; 46.25)**	28 (17.75; 53.75)
	**GAA**	5 (0; 22.75)	**25 ^{AB} (17.25; 30.5)**	30.5 (17.25; 38)	24.5 (13.75; 47.25)	30.5 (19; 48.75)	**33.5 ^{AB} (25.75; 47)**	**15.5 ^{B} (9.75; 22.5)**	15 (5.25; 34.5)
	**GC**	**32 ^{ab} (0.75; 49.5)**	**4.5 ^{bB} (0.75; 21.25)**	**2 ^{b} (0; 21.5)**	**10 ^{ab} (2.25; 24.75)**	**51.5 ^{a} (29.25; 57.25)**	**47 ^{aA} (44.5; 54)**	**42.5 ^{abA} (25.75; 48.25)**	**31.5 ^{ab} (18.75; 39.25)**
	**GCA**	18.5 (1.75; 29.5)	**29 ^{A} (22.75; 34.25)**	34 (23.25; 45)	46.5 (29.5; 60.25)	34.5 (23.75; 52.5)	**24 ^{B} (3.75; 48.75)**	**26.5 ^{AB} (9.75; 38.5)**	28.5 (18; 33.5)
**Stay at the back of the stall^{d}**	**GA**	0 (0; 0)	0 (0; 0)	0 (0; 0)	0 (0; 0)	0 (0; 1.75)	0 (0; 3.5)	0 (0; 0.25)	0 (0; 0)
	**GAA**	0 (0; 0.25)	0 (0; 0)	0 (0; 0.5)	0 (0; 0)	0 (0; 0)	0 (0; 0)	0 (0; 0)	0 (0; 0.25)
	**GC**	0 (0; 9.5)	0 (0; 0)	0 (0; 4.5)	0 (0; 5)	0 (0; 0)	0 (0; 4.5)	0 (0; 9.75)	3.5 (0; 25)
	**GCA**	0 (0; 0.25)	0 (0; 0)	0 (0; 0)	0 (0; 0)	0 (0; 0)	0 (0; 0.25)	1 (0; 7.25)	0 (0; 5.5)
**Look at the back of the stall^{d}**	**GA**	0 (0; 0)	0 (0; 0)	**0 ^{B} (0; 0)**	0 (0; 0)	0 (0; 0.25)	0 (0; 0)	0 (0; 0.25)	**0 ^{B} (0; 0)**
	**GAA**	0 (0; 0.5)	0 (0; 0)	**0 ^{AB} (0; 3)**	0 (0; 0)	0 (0; 2.25)	0 (0; 0)	0 (0; 0)	**0.5 ^{AB} (0; 2.25)**
	**GC**	2 (0; 29.75)	0 (0; 2.5)	**1.5 ^{A} (0; 18.5)**	1 (0; 19)	0 (0; 1)	0 (0; 0.75)	2.5 (0; 22.25)	**2 ^{A} (0; 3.5)**
	**GCA**	0 (0; 5)	0 (0; 0)	**0 ^{B} (0; 0)**	0 (0; 0)	0 (0; 0.25)	0 (0; 1)	0 (0; 1.5)	**0 ^{AB} (0; 0.25)**
**Look at the wound^{f}**	**GA**	0 (0; 0)	0 (0; 0)	**0 ^{B} (0; 0)**	0 (0; 0)	0 (0; 0)	0 (0; 0)	0 (0; 1.25)	0 (0; 0)
	**GAA**	0 (0; 0)	0 (0; 0)	**0 ^{B} (0; 0)**	0 (0; 0)	0 (0; 0)	0 (0; 0)	0 (0; 0)	0 (0; 0)
	**GC**	0 (0; 0)	0 (0; 4.25)	**1 ^{A} (0; 7.75)**	0 (0; 3.5)	0 (0; 2.75)	0 (0; 1.25)	0 (0; 0.5)	0 (0; 0)
	**GCA**	0 (0; 0)	0 (0; 0)	**0 ^{B} (0; 0)**	0 (0; 2.75)	0 (0; 0.5)	0 (0; 0)	0 (0; 0)	0 (0; 0)
**Retract pelvic limb^{f}**	**GA**	0 (0; 0)	0 (0; 0)	0 (0; 0)	**0 ^{B} (0; 0)**	**0 ^{B} (0; 0)**	0 (0; 0.25)	0 (0; 0.25)	**0 ^{B} (0; 0)**
	**GAA**	0 (0; 0)	0 (0; 0)	0 (0; 0)	**0 ^{B} (0; 0)**	**0 ^{B} (0; 0)**	0 (0; 0)	0 (0; 0)	**0 ^{B} (0; 0)**
	**GC**	0 (0; 0)	0 (0; 0)	0 (0; 4)	**3 ^{A} (0; 12)**	**1.5 ^{A} (0; 12)**	0 (0; 13.25)	0 (0; 0)	**1 ^{A} (0; 2)**
	**GCA**	0 (0; 0)	0 (0; 0)	0 (0; 0)	**0 ^{AB} (0; 1.25)**	**0 ^{AB} (0; 0.75)**	0 (0; 0)	0 (0; 0.25)	**0 ^{B} (0; 0.25)**
**Expose the penis^{f}**	**GA**	**0 ^{B} (0; 0)**	0 (0; 0)	0 (0; 0)	0 (0; 0)	0 (0; 0.25)	0 (0; 0.5)	0 (0; 0.75)	0 (0; 0)
	**GAA**	**0 ^{B} (0; 0.25)**	0 (0; 0.25)	0 (0; 0.25)	0 (0; 2.25)	0 (0; 0)	0 (0; 0)	0 (0; 0)	0 (0; 0.75)
	**GC**	**1 ^{AB} (0; 3)**	0 (0; 0)	0 (0; 0)	0 (0; 0.5)	0 (0; 1.25)	0 (0; 4.5)	0 (0; 1.5)	1.5 (0; 4.25)
	**GCA**	**2 ^{A} (1.75; 3)**	0 (0; 1.5)	0 (0; 2.25)	0 (0; 8)	2.5 (0; 10.5)	1.5 (0; 2.75)	0 (0; 0.25)	0.5 (0; 3.75)
