# Supplementary figures and images for: Spontaneous Behaviors of Post-Orchiectomy Pain in Horses Regardless of the Effects of Time of Day, Anesthesia, and Analgesia
Source: Animals (Basel). 2021 May 31;11(6):1629. doi: 10.3390/ani11061629 (PMC8230028; doi:10.3390/ani11061629)

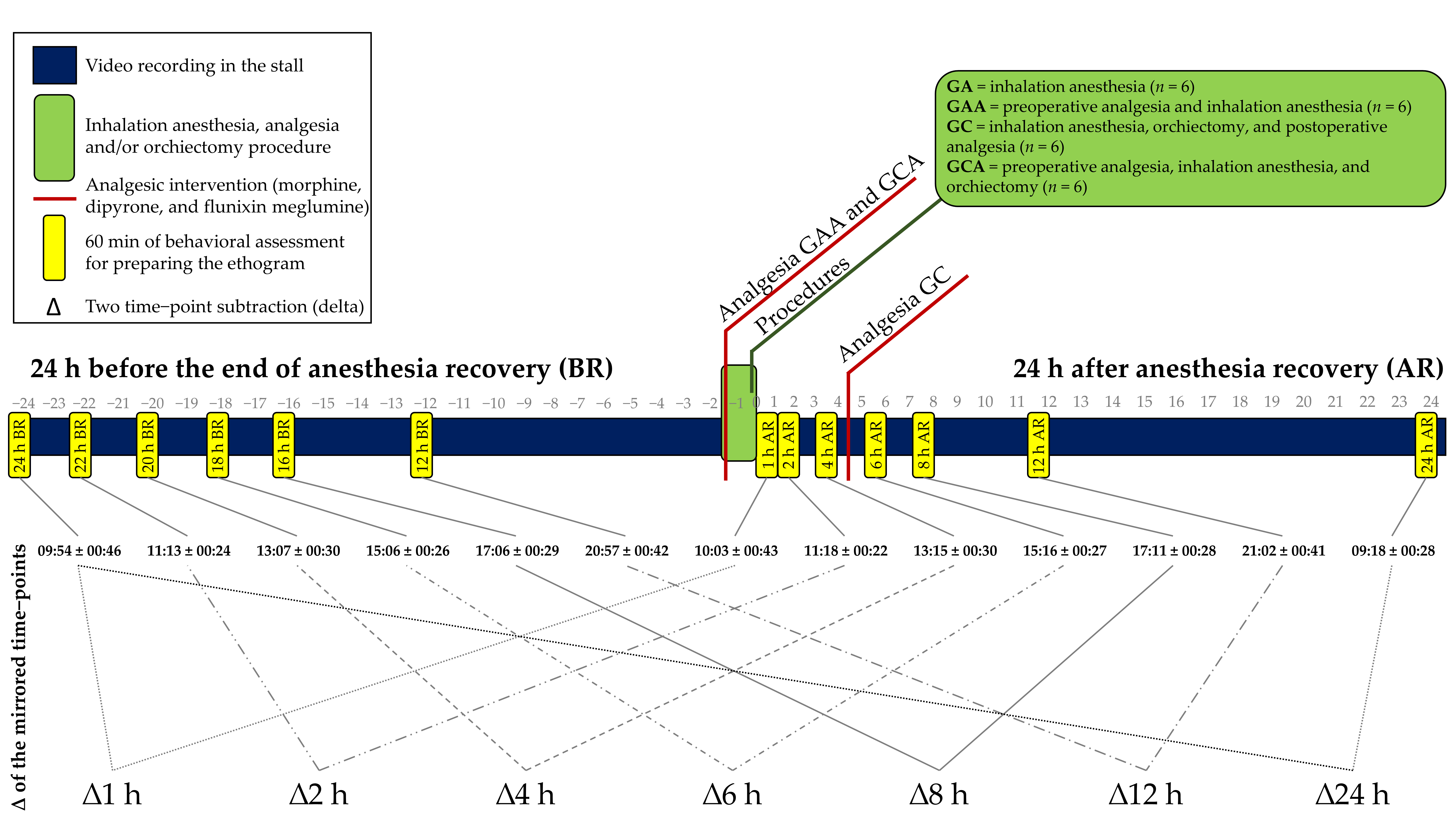

Supplement: Supplementary file 1 [file animals-11-01629-s001.zip › Figure1_final.tif]

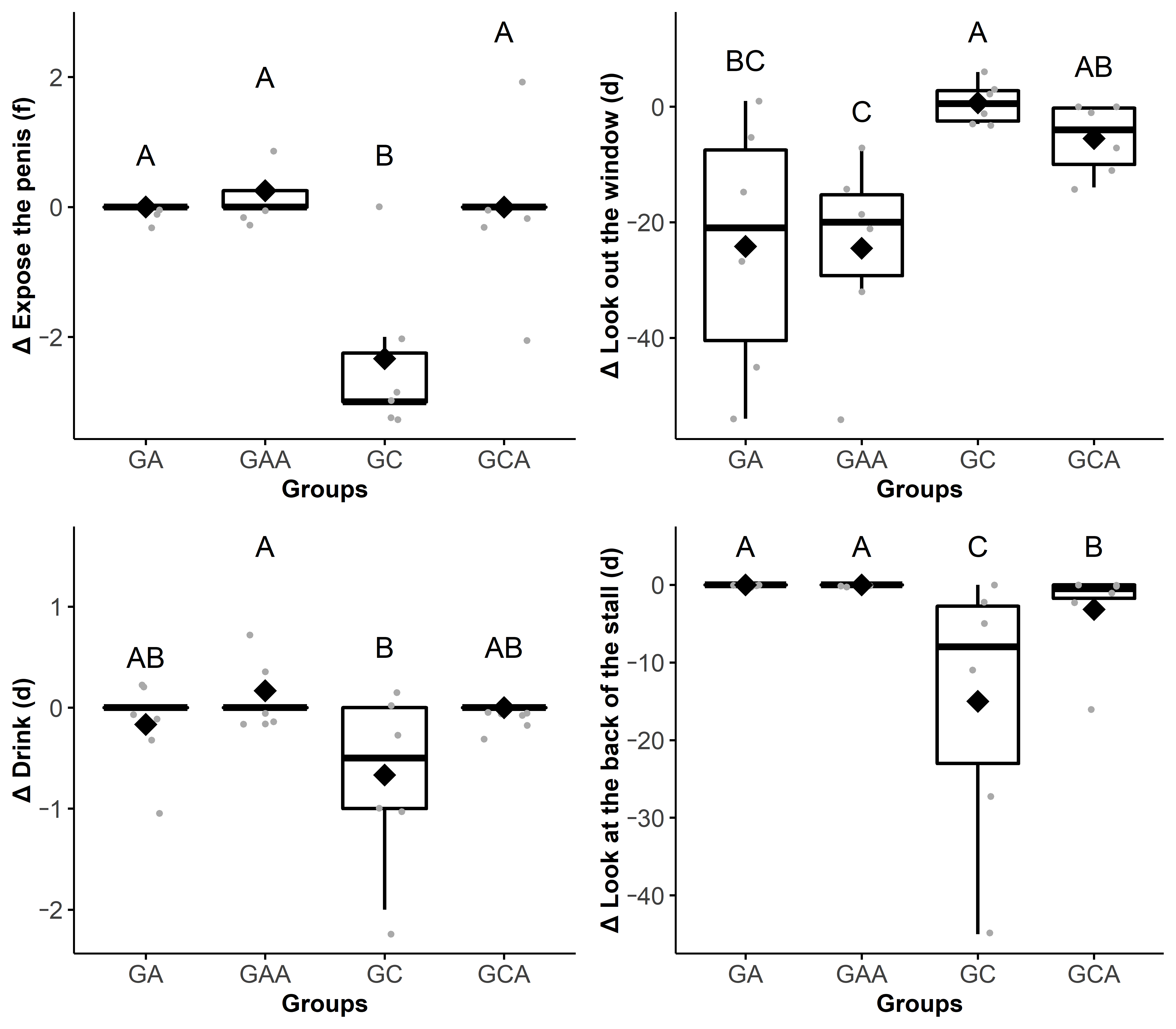

Supplement: Supplementary file 1 [file animals-11-01629-s001.zip › Figure2_final.tif]

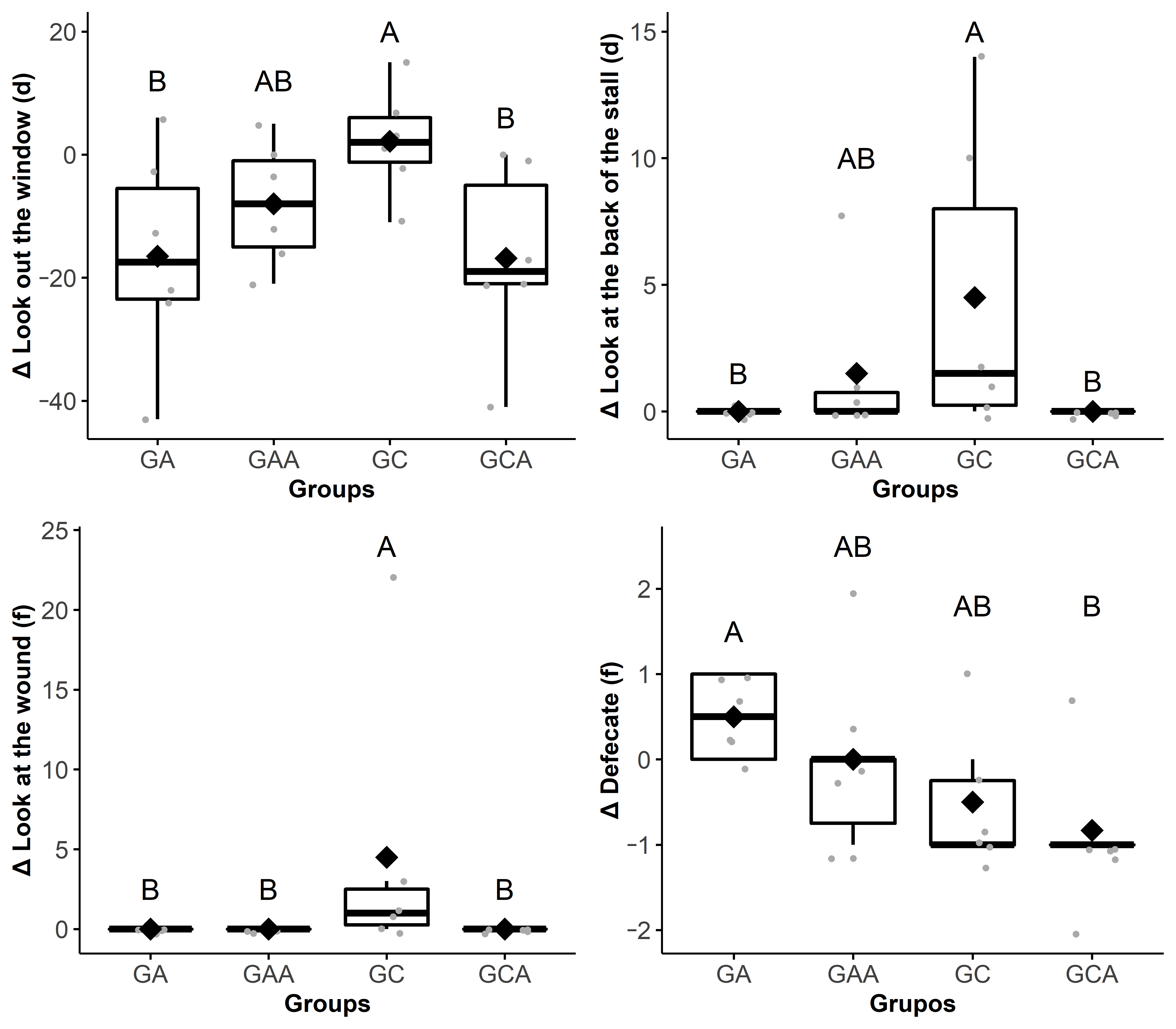

Supplement: Supplementary file 1 [file animals-11-01629-s001.zip › Figure3_final.tif]

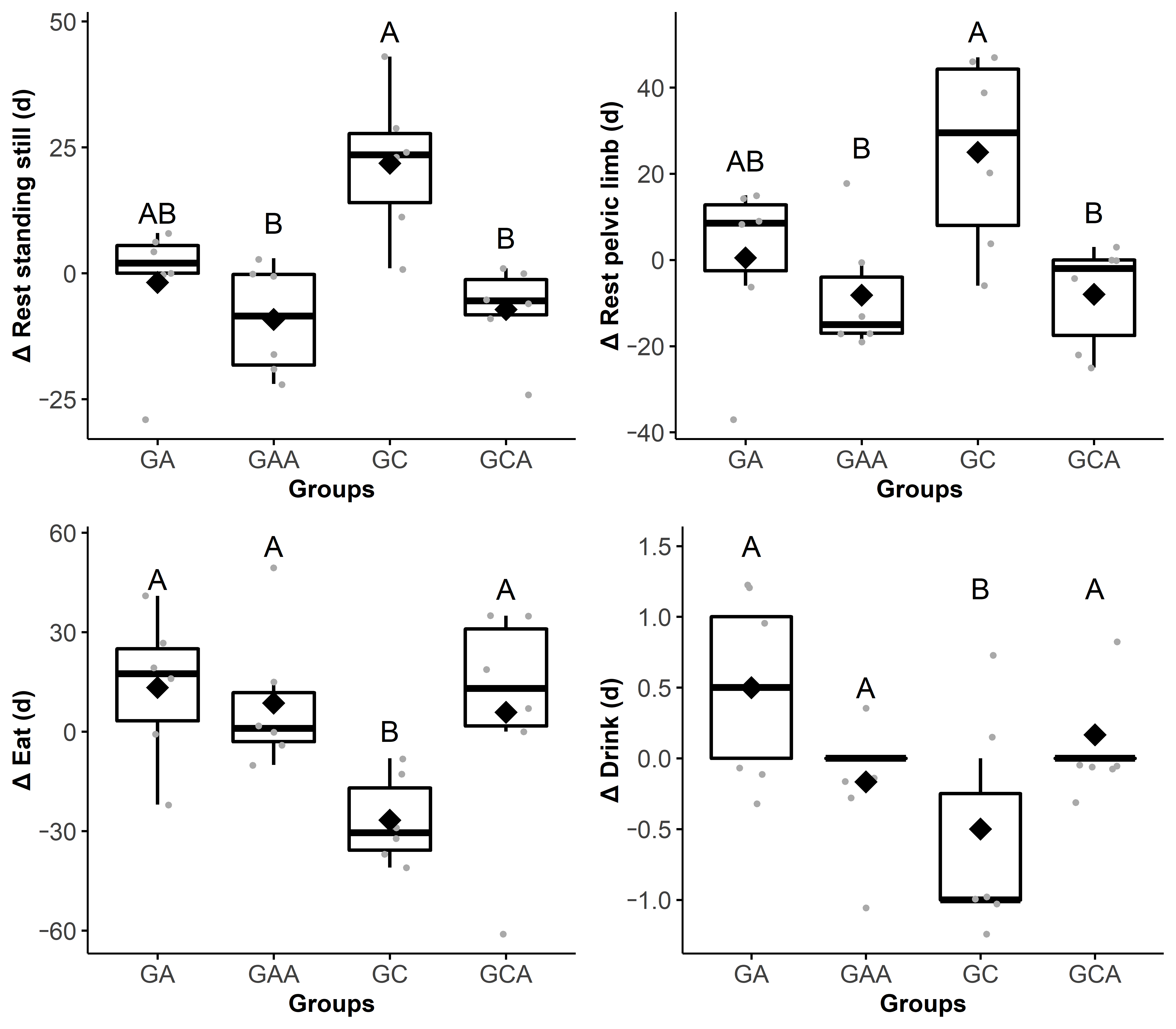

Supplement: Supplementary file 1 [file animals-11-01629-s001.zip › Figure4_final.tif]

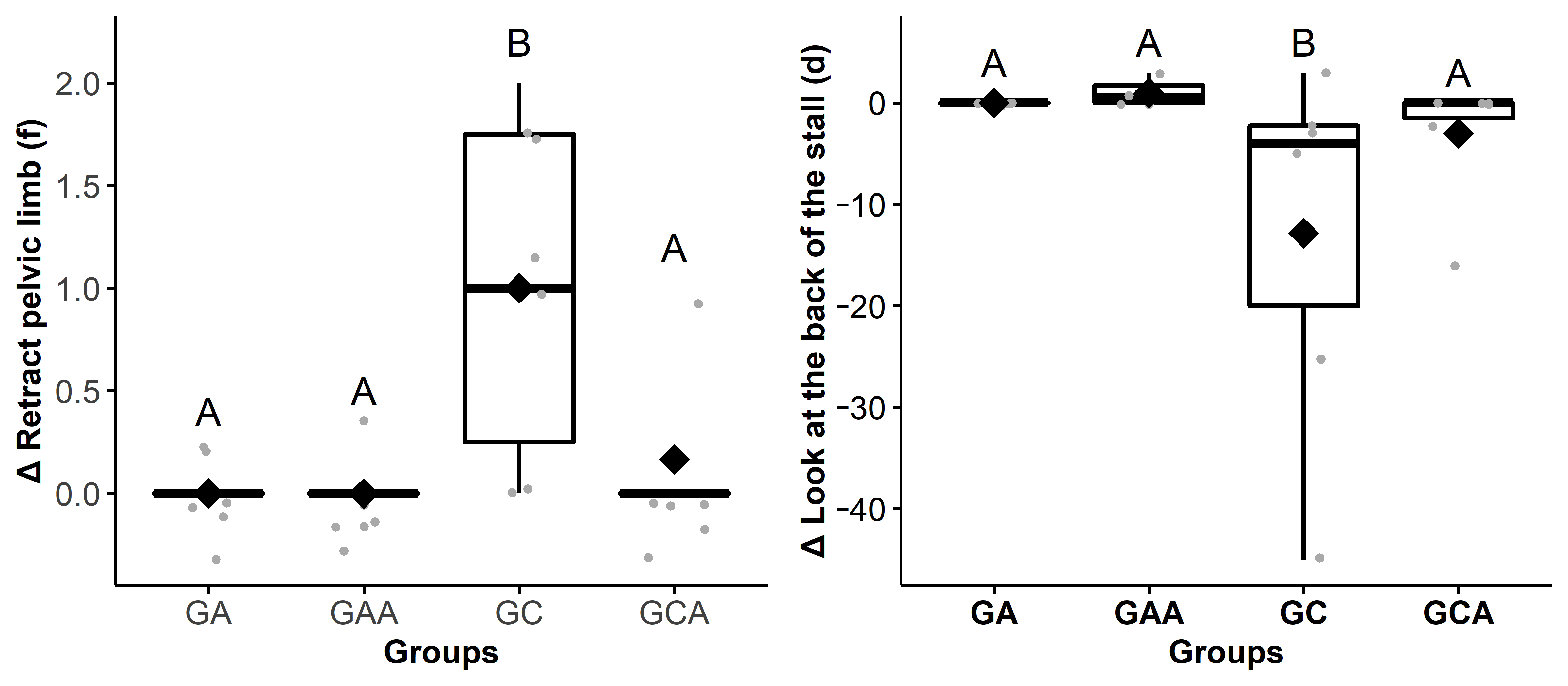

Supplement: Supplementary file 1 [file animals-11-01629-s001.zip › Figure5_final.tif]
